# GAADE: identification spatially variable genes based on adaptive graph attention network

**DOI:** 10.1093/bib/bbae669

**Published:** 2024-12-20

**Authors:** Tianjiao Zhang, Hao Sun, Zhenao Wu, Zhongqian Zhao, Xingjie Zhao, Hongfei Zhang, Bo Gao, Guohua Wang

**Affiliations:** College of Computer and Control Engineering, Northeast Forestry University, No. 26 Hexing Road, Xiangfang District, Harbin 150040, China; College of Computer and Control Engineering, Northeast Forestry University, No. 26 Hexing Road, Xiangfang District, Harbin 150040, China; College of Computer and Control Engineering, Northeast Forestry University, No. 26 Hexing Road, Xiangfang District, Harbin 150040, China; College of Computer and Control Engineering, Northeast Forestry University, No. 26 Hexing Road, Xiangfang District, Harbin 150040, China; College of Computer and Control Engineering, Northeast Forestry University, No. 26 Hexing Road, Xiangfang District, Harbin 150040, China; College of Computer and Control Engineering, Northeast Forestry University, No. 26 Hexing Road, Xiangfang District, Harbin 150040, China; Department of Radiology, The Second Affiliated Hospital of Harbin Medical University, No. 246 Xuefu Road, Nangang District, Harbin 150081, China; College of Computer and Control Engineering, Northeast Forestry University, No. 26 Hexing Road, Xiangfang District, Harbin 150040, China; Faculty of Computing, Harbin Institute of Technology, No. 92 West Da Zhi Street, Nangang District, Harbin 150001, China

**Keywords:** ST-seq, spatially variable gene, spatial domain, graph attention auto-encoders, spatial neighbor graph

## Abstract

The rapid advancement of spatial transcriptomics (ST) sequencing technology has made it possible to capture gene expression with spatial coordinate information at the cellular level. Although many methods in ST data analysis can detect spatially variable genes (SVGs), these methods often fail to identify genes with explicit spatial expression patterns due to the lack of consideration for spatial domains. Considering spatial domains is crucial for identifying SVGs as it focuses the analysis of gene expression changes on biologically relevant regions, aiding in the more accurate identification of SVGs associated with specific cell types. Existing methods for identifying SVGs based on spatial domains predefine spot similarity before training, which prevents adaptive learning and limits generalizability across different tissues or samples. This limitation may also lead to inaccurate identification of specific genes at boundary regions. To address these issues, we present GAADE, an unsupervised neural network architecture based on graph-structured data representation learning. GAADE stacks encoder/decoder layers and integrates a self-attention mechanism to reconstruct node attributes and graph structure, effectively capturing spatial domain structures of different sections. Consequently, we confine the identification of SVGs within spatial domains. By performing differential expression analysis on spots within the target spatial domain and their multi-order neighbors, GAADE detects genes with enriched expression patterns within defined domains. Comparative evaluations with five other popular methods on ST datasets across four different species, regions and tissues demonstrate that GAADE exhibits superior performance in detecting SVGs and capturing the extent of spatial gene expression variation.

## Introduction

Although single-cell RNA sequencing (scRNA-Seq) technology enables the study of cellular heterogeneity and cell type classification, its inability to provide spatial information limits systematic research on the relationship between the physiological structure and function of various tissues and organs [1]. To address this limitation, spatial transcriptomics (ST), and other spatial omics methods have been developed. In the context of tissues, understanding the spatial patterns of gene expression is crucial for comprehensively understanding disease states, tissue development and function, which is made possible through the application of ST. Advances in ST technology allow us to analyze gene expression profiles within their spatial context, providing unprecedented insights into how gene expression in cells is influenced by their surrounding environment [2–4]. By employing ST techniques, gene expression profiles can be effectively integrated with tissue structures, adding a novel and essential dimension to data interpretation, thereby providing a more comprehensive understanding of biological and pathological mechanisms.

A common task in all ST analyses is to identify genes with spatial expression patterns, referred to as spatially variable genes (SVGs). These genes often exhibit non-random expression in specific tissue regions, contributing to processes such as developmental gradients, cell signaling pathways and the tumor microenvironment [5–8]. The inference of SVGs also facilitates the systematic analysis of cell states, intercellular communication and the identification of key phenotypes and functions within an organism [9]. With advances in bioinformatics in the field of ST, several emerging computational methods, primarily developed in R or Python, have been proposed for identifying SVGs. Among these, statistical model-based methods are predominant, such as trendsceek [10], SpatialDE [11], and SPARK [12]. These methods utilize known gene expression profiles and spatial information of cells to construct a statistical framework that determines the dependency between gene expression values and the spatial positions of cells, yielding a p-value to indicate the spatial variability of gene expression. Similar to trendsceek, the ScGCO [13] (single-cell graph cuts optimization) method also conceptualizes gene expression data as a marked point process. This approach evaluates the dependency of points exhibiting a specific mark on their spatial locations through hypothesis testing. Under the absence of spatial dependency, it posits that points with a specific mark in a 2D space are distributed randomly, conforming to a homogeneous spatial Poisson process. Genes are classified as SVGs if their corresponding spatial regions exhibit a statistically significant low probability of specific marks under the null model. However, the parameter inference strategies employed by these methods may encounter several issues, such as susceptibility to local optima and sensitivity to errors in prior assumptions. Beyond these technical challenges, statistical modeling approaches rely solely on statistical significance to assess spatial gene expression patterns, making it difficult to interpret the spatial heterogeneity and homogeneity of SVGs from a spatial perspective. In addition, In the realm of Gaussian process methodologies, the selection of the kernel function presents a significant challenge. While [14] introduces improvements by enabling gene-specific kernel parameter selection, it remains constrained to a single type of kernel function. This limitation underscores the necessity for further methodological innovations aimed at optimizing kernel selection in the context of SVG detection.

Considering the rich features and well-structured input data, neural networks—an important branch of machine learning—are widely used for analyzing scRNA-seq and ST data [15, 16], as seen in methods like SOMDE [17] and SPADE [18]. SOMDE leverages Self-Organizing Map (SOM) neural networks and Gaussian processes to model spatial data, enabling efficient identification of SVGs. The runtime of SOMDE is related to the number of features, and in high-dimensional, complex feature data, it often leads to dimensional redundancy and processing challenges. SPADE, on the other hand, utilizes imaging data and ST data as inputs, extracting morphological features around each spot through a convolutional neural network and combining them with gene expression data to identify key genes associated with spatial and morphological heterogeneity. However, due to the reliance on image data, SPADE’s performance may vary depending on the scale of image segmentation and the density of the captured spots. Additionally, grid-based spatial methods analyze spatial gene expression by dividing spatial grids or constructing adjacency matrices, as exemplified by SingleCellHaystack [19] and MERINGUE [20]. SingleCellHaystack divides the spatial area into grids and determines grid points based on cell density. Cells are classified into two categories depending on whether a gene can be detected. SingleCellHaystack then calculates the distribution of these two categories and compares it with the random distribution of cells in space. It uses Kullback–Leibler divergence to calculate a D_KL_ score for each gene, identifying genes with uneven expression in multidimensional space, and evaluates the spatial variability of the gene based on this score. MERINGUE, on the other hand, employs Delaunay triangulation to consider each cell as a neighborhood and determines the adjacency relationship between pairs of cells based on these neighborhoods, representing this relationship with a binary adjacency weight matrix. Based on the adjacency matrix and gene expression matrix, MERINGUE calculates Moran’s I statistic to identify significant spatial genes. A common limitation of grid-based spatial methods is that they binarize gene expression values rather than providing them in continuous observation form. Additionally, selecting an appropriate threshold requires considerable time. Although existing methods consider the spatial structure of ST data, they typically predefine fixed similarity relationships between neighboring spots prior to training, which are not obtained through adaptive learning. Recent SVG detection methods employ model-free techniques to identify SVGs. The BSP (Big-Small Patch) [21] method, recently introduced in a publication, employs a non-parametric model to identify SVGs in 2D or 3D ST data. This approach utilizes normalized ST data as input and defines big and small patches for each spatial spot based on neighboring spots with larger or smaller radis, respectively. It subsequently computes the local means of gene expression for both big and small patches. The method then assesses the ratio of the variances of these local means for each gene, approximating a log-normal distribution for these ratios. Finally, a p-value is calculated for each gene based on this approximated distribution.

To this end, we have developed a deep learning model based on a graph attention auto-encoder for identifying SVGs, named GAADE. This model utilizes a graph attention auto-encoder to learn low-dimensional latent embeddings that incorporate spatial information and gene expression data, constructing a spatial neighbor network to capture spatial domain information. Through adaptive learning, GAADE effectively identifies spatially specific gene expression in small functional regions within tissues and provides a comprehensive view of gene expression gradients across the tissue. We approach the tasks of spatial domain partitioning and SVGs identification as an integrated problem. GAADE employs an adaptive graph attention auto-encoder to learn low-dimensional latent embeddings and builds a spatial neighbor network that incorporates gene expression and spatial location data to better characterize spatial similarity [22]. The attention mechanism adaptively learns edge weights in the spatial neighbor network and uses them to aggregate neighbor information to update point representations. Subsequently, UMAP is used for data visualization, and clustering algorithms identify spatial domains with coherent expression and histological features, ensuring that detected SVGs exhibit spatial expression patterns within defined spatial domains [23]. Extensive testing across multiple ST platforms demonstrates that GAADE excels in tasks such as spatial domain delineation, SVGs identification, and spatial trajectory inference.

## Materials and methods

### Datasets

All datasets used in this study are publicly available and can be downloaded. We used spatial gene expression datasets from four different tissues (Table 1).

**Table 1:** Description of the datasets used in the study.

Dataset	Spots	Genes	Domains	Protocol	Species
DLPFC_151507	4221	33,538	7	10X Visium	*Homo sapiens*
DLPFC_151508	4381	33,538	7	10X Visium	*H. sapiens*
DLPFC_151509	4788	33,538	7	10X Visium	*H. sapiens*
DLPFC_151510	4595	33,538	7	10X Visium	*H. sapiens*
DLPFC_151,669	3636	33,538	5	10X Visium	*H. sapiens*
DLPFC_151,670	3484	33,538	5	10X Visium	*H. sapiens*
DLPFC_151,671	4093	33,538	5	10X Visium	*H. sapiens*
DLPFC_151,672	3888	33,538	5	10X Visium	*H. sapiens*
DLPFC_151,673	3611	33,538	7	10X Visium	*H. sapiens*
DLPFC_151,674	3635	33,538	7	10X Visium	*H. sapiens*
DLPFC_151675	3566	33,538	7	10X Visium	*H. sapiens*
DLPFC_151676	3431	33,538	7	10X Visium	*H. sapiens*
Breast Cancer	3798	36,601	–	10X Visium	*H. sapiens*
Brain coronal	2903	32,285	–	10X Visium	*Mus musculus*
Brain (Sagittal-Anterior)	2695	32,285	–	10X Visium	*M. musculus*

The first dataset consists of 12 tissue sections of the human dorsolateral prefrontal cortex (DLPFC) collected using 10X Visium (http://research.libd.org/spatialLIBD/). Specifically, the DLPFC dataset was sampled from three experimental subjects, with the number of spots per section ranging from 3498 to 4789, capturing 33,538 genes. Each section is manually annotated and includes five to seven regions, namely the DLPFC layers and white matter.

The second dataset is a mouse brain tissue dataset downloaded from the public 10x Genomics data repository (https://cf.10xgenomics.com/samples/spatial-exp/1.1.0/V1_Mouse_Brain_Sagittal_Anterior/). This dataset comprises two sections, from which we selected the anterior sagittal section. The selected section contains 2695 spots, capturing 21,334 genes.

The third dataset is a human breast cancer sample, including ductal carcinoma in situ (DCIS) and invasive carcinoma. The selected section contains 2518 spots, capturing 17 651 genes. This dataset is available on the 10x Genomics website (https://www.10xgenomics.com/resources/datasets).

The fourth dataset is a selected section of an adult mouse coronal brain containing 2903 spots, capturing 21 747 genes. This dataset can be obtained from https://www.10xgenomics.com/datasets/adult-mouse-brain-section-1-coronal-stains-dapi-anti-neu-n-1-standard-1-1-0.

### Overview of the GAADE

**Figure 1:**
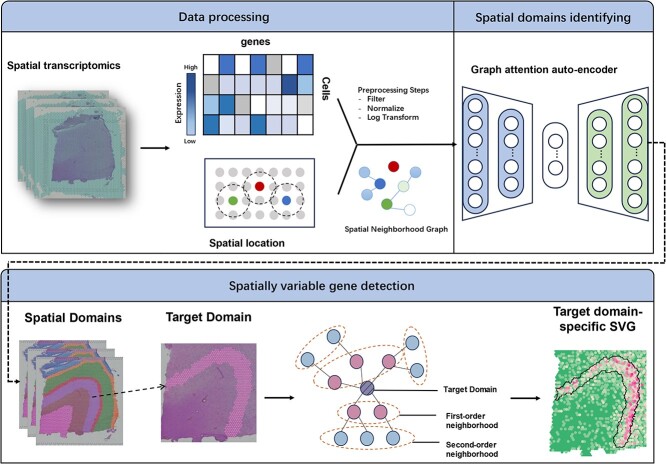
Workflow of GAADE.

The key advantage of GAADE is its ability to integrate gene expression with spatial information to gain deep insights into the structure, functional organization and expression patterns. GAADE constructs a spatial neighbor graph (SNG) based on the spatial proximity of spots, connecting physically adjacent cells, and enabling neighborhood-based analysis (Fig. 1). It employs a graph attention auto-encoder to learn low-dimensional latent embeddings that capture both spatial and gene expression data, learning node representations in an unsupervised manner. The autoencoder, with stacked encoder/decoder layers, reconstructs node features using the graph structure and aggregates neighbor information to identify spatial domains and quantify boundary differences. To identify SVGs, GAADE performs differential expression (DE) analysis on spots within each spatial domain and their neighbors, identifying genes with adjusted p-values below 0.05 as SVGs.

### Data preprocessing

For all datasets, spots outside the main tissue regions were removed, along with exogenous RNA, mitochondrial genes and genes expressed in fewer than three spots. Gene expression counts were log-transformed and normalized using SCANPY by dividing each gene’s UMI count by the total UMI count per spot [24]. The top 3000 highly variable genes were then selected to learn latent representations of cells and spots.

### Graph construction for ST data

To integrate the similarity of neighboring points for a given spot, GAADE first constructs a spatial neighbor graph. The method takes into account that aggregating the adjacency information of each spot can enhance the representational capacity of spatial patterns, thereby strengthening the identification of SVGs with specificity. we construct the SNG based on the spatial coordinates of each spot in Euclidean space to determine the k-nearest neighbors for each spot [25]. Specifically, in this graph, each vertex *v*∈*V* represents a specific spot and each pair of vertices in *V* is connected by an edge with a calculated weight. The weights *W* of the SNG are defined by Equation (1):


(1) \begin{equation*} {w}_{ij}=\left\{\begin{array}{@{}ll}1-\displaystyle\frac{S_{ij}}{\max \left({\mathbf{S}}_{i.}\right)},&\ \mathrm{if}\ \mathrm{spots}\ i\ \mathrm{and}\ j\ \mathrm{are}\ \mathrm{neighbors}\ \\{}0,&\ \mathrm{otherwise}\ \end{array}\right., \end{equation*}

In the above equation, *W_ij_* represents the weight between spot *i* and spot *j*, *S_ij_* denotes the distance between spot *i* and spot *j*, and max(*S_i_.*​) indicates the maximum distance between spot *i* and its nearest neighbor. Spots are considered spatial neighbors if the weight between two spots is less than the rad_cutoff value. By adjusting this value, we ensure that each spot has an average of six spatial neighbors, thereby balancing the aggregation of local spatial information with computational complexity.

### Graph attention auto-encoders

#### Encoder

In our architecture, the encoder takes normalized gene expression data as input and generates node representations through the graph structure by utilizing node features and stacking layers. These representations are then propagated through the graph structure, resulting in richer node embeddings. Each encoder layer generates new node representations based on the relevance of neighboring nodes’ representations. We employ a self-attention mechanism with shared parameters among nodes. In the *k*-th layer of the encoder, the relevance between neighboring node *i* and node *j* is computed as follows:


(2) \begin{equation*} {e}_{ij}^{(k)}=\mathrm{Sigmoid}\left({\mathbf{v}}_s^{(k)^T}\sigma \left({\mathbf{W}}^{(k)}{\mathbf{h}}_i^{\left(k-1\right)}\right)+{\mathbf{v}}_r^{(k)^T}\sigma \left({\mathbf{W}}^{(k)}{\mathbf{h}}_j^{\left(k-1\right)}\right)\right), \end{equation*}

Here, ${\mathbf{W}}^{(k)}\in{\mathbb{R}}^{d^{(k)}\times{d}^{\left(k-1\right)}}$, ${\mathbf{v}}_s^{(k)}\in{\mathbb{R}}^{d^{(k)}}$, ${\mathbf{v}}_r^{(k)}\in{\mathbb{R}}^{d^{(k)}}$ represents the trainable parameters of the *k*-th encoder layer, and $\sigma$ denotes the activation function [26]. Sigmoid represents the Sigmoid function (Sigmoid(*x*) = 1/(1 + *e*^-*x*^)).

To make the relevance scores of the neighbors of node *i* comparable, we normalize them using the softmax function, as follows:


(3) \begin{equation*} {\alpha}_{ij}^{(k)}=\frac{\exp \left({e}_{ij}^{(k)}\right)}{\sum_{l\in{N}_i}\kern0.1em \exp \left({e}_{il}^{(k)}\right)}, \end{equation*}

where *N_i_* represents the neighborhood of node *i* (i.e. the set of nodes connected to node *i* according to the adjacency matrix A, including node *i* itself).

By treating the node features as the initial node representations (i.e. *h*_i_^(0)^ = *x_i_* ​, ∀ *i*∈{1,2,…,*N*}, the *k*-th layer of the encoder generates the representation of node *i* at the *k*-th layer as follows:


(4) \begin{equation*} {\mathbf{h}}_i^{(k)}=\sum_{j\in{\mathcal{N}}_i}\kern0.1em {\alpha}_{ij}^{(k)}\sigma \left({\mathbf{W}}^{(k)}{\mathbf{h}}_j^{\left(k-1\right)}\right), \end{equation*}

After applying *L* encoder layers, we consider the output of the final layer as the ultimate node representation.

#### Decoder

In contrast to the encoder, the decoder reverses the latent embeddings into reconstructed normalized expression profiles. Treating the output of the encoder as the input to the decoder, the encoding process is reversed to learn node representations without any supervision. Each decoder layer reconstructs the node representations based on the relevance of neighboring nodes’ representations. In the *k*-th decoder layer, the normalized relevance (i.e. attention coefficient) between node *j* and its neighboring node *i* is calculated as follows:


(5) \begin{equation*} \kern-9.5pc{\hat{\alpha}}_{ij}^{(k)}=\frac{\exp \left({\hat{e}}_{ij}^{(k)}\right)}{\sum_{l\in{\mathcal{N}}_i}\kern0.1em \exp \left({\hat{e}}_{il}^{(k)}\right)}, \end{equation*}

(6) \begin{equation*} {\hat{e}}_{ij}^{(k)}=\mathrm{Sigmoid}\left({\hat{\boldsymbol{v}}}_s^{(k)^T}\sigma \left({\hat{\boldsymbol{W}}}^{(k)}{\hat{\boldsymbol{h}}}_i^{(k)}\right)+{\hat{\boldsymbol{v}}}_r^{(k)^T}\sigma \left({\hat{\boldsymbol{W}}}^{(k)}{\hat{\boldsymbol{h}}}_j^{(k)}\right)\right), \end{equation*}

Here, ${\hat{\mathbf{W}}}^{(k)}\in{\mathbb{R}}^{d^{\left(k-1\right)}\times{d}^{(k)}}$, ${\hat{\mathbf{v}}}_s^{(k)}\in{\mathbb{R}}^{d^{\left(k-1\right)}}$ and ${\hat{\mathbf{v}}}_r^{(k)}\in{\mathbb{R}}^{d^{\left(k-1\right)}}$ represents the trainable parameters of the *k*-th decoder layer.

Treating the output of the encoder as the input to the decoder, the *k*-th layer of the decoder reconstructs the representation of node *i* at the layer *k*-1 as follows:


(7) \begin{equation*} {\hat{\mathbf{h}}}_i^{\left(k-1\right)}=\sum_{j\in{\mathcal{N}}_i}\kern0.1em {\hat{\alpha}}_{ij}^{(k)}\sigma \left({\hat{\mathbf{W}}}^{(k)}{\hat{\mathbf{h}}}_j^{(k)}\right), \end{equation*}

After applying *L* decoder layers, we consider the output of the final layer as the reconstructed node features.

### Loss function

Both node features and the graph structure should be encoded by high-quality node representations. We minimize the reconstruction loss of node features using the following method:


(8) \begin{equation*} \sum_{i=1}^N\kern0.1em {\| {\mathbf{x}}_i-{\hat{\mathbf{x}}}_i\|}_2, \end{equation*}

We minimize the reconstruction loss of the node features and the graph structure as follows:


(9) \begin{equation*} \mathrm{Loss}=\sum_{i=1}^N\kern0.1em {\| {\mathbf{x}}_i-{\hat{\mathbf{x}}}_i\|}_2-\lambda \sum_{j\in{\mathcal{N}}_i}\kern0.1em \log \left(\frac{1}{1+\exp \left(-{\mathbf{h}}_i^T{\mathbf{h}}_j\right)}\right), \end{equation*}

Here, *λ* controls the contribution of the graph structure reconstruction loss.

### Spatial domain assignment by clustering

After model training, we use the non-spatial clustering algorithm Mclust to cluster the spatial gene expression data reconstructed by the decoder [27]. This groups spots into distinct spatial domains with similar gene expression profiles and spatial proximity. For manually annotated tissue slices, we set the number of clusters to be the same as the actual labels. For tissue slices without prior information, we determine the number of clusters by trying different values.

### Refinement of spatial domain module

For ST data with low spatial resolution, we developed a refined spatial domain boundary module using an attention mechanism, suitable for processing data from 10x Visium technology. This module enhances the detection of spatial heterogeneity at domain boundaries but should be cautiously applied to data with cellular or subcellular resolution due to potential noise. Adjusting the hyperparameter α is critical; larger values may reduce the flexibility of the graph attention mechanism, impacting SVG detection. Thus, α can be tuned to optimize performance based on specific research needs.

To select genes with enriched expression in the target domain, we apply strict filtering for genes with positive spatial autocorrelation coefficients and adjusted p-values below 0.05. The ratio of the total expression spots within the target domain to the observed spots must exceed 80%, indicating higher expression density in the target domain compared to other regions. The average gene expression within the target domain is then calculated using the following formula:


(10) \begin{equation*} {\mathrm{Expresion}}_{ij}=\frac{{\mathrm{count}}_{ij}}{\sum_j\kern0.1em {\mathrm{count}}_{ij}}\times 100\%, \end{equation*}

where *i* represents cell *i* and *j* represents gene *j*.

GAADE employs the Wilcoxon rank-sum test for DE analysis between the target domain and its first-order and second-order neighborhoods. It calculates the percentage difference by comparing the average gene expression in the target domain with those in the neighboring domains, ensuring that the target domain’s expression is higher. This approach allows for a comprehensive understanding of spatial characteristics and patterns around the target domain.

### Evaluation metrics

Spatial autocorrelation statistics are widely used in spatial data analysis tools to assess the spatial autocorrelation of continuous features. In ST data, gene expression values at local spatial neighborhood points tend to be closer to each other than those at distant points. Therefore, genes with strong spatial autocorrelation can exhibit more organized spatial expression patterns. To evaluate whether the detected SVGs display organized spatial expression patterns, we employed Moran’s I [28] and Geary’s C [29], two commonly used statistical methods, to quantify the degree of spatial autocorrelation in gene expression. For a given gene, these metrics measure the similarity of a point to its surrounding points. The range of Moran’s I values is from −1 to 1, where values close to 1 indicate a clear spatial pattern, values close to 0 suggest random spatial expression, and values near −1 indicate a disorganized spatial pattern. For each gene, the Moran’s I score is calculated as follows:


(11) \begin{equation*} I=\frac{N}{W}\frac{\sum_i\kern0.1em {\sum}_j\kern0.1em \left[{w}_{ij}\left({x}_i-\overline{x}\right)\left({x}_j-\overline{x}\right)\right]}{\sum_i\kern0.1em {\left({x}_i-\overline{x}\right)}^2}, \end{equation*}

Geary’s C is another commonly used statistic that is inversely related to Moran’s I, though it is not identical. Unlike Moran’s I, which measures global spatial autocorrelation, Geary’s C is more sensitive to local spatial autocorrelation. The formula for calculating Geary’s C score for each gene is as follows:


(12) \begin{equation*} C=\frac{\left(N-1\right){\sum}_i\kern0.1em {\sum}_j\kern0.1em \left[{w}_{ij}{\left({x}_i-{x}_j\right)}^2\right]}{2W{\sum}_i\kern0.1em {\left({x}_i-\overline{x}\right)}^2}, \end{equation*}

### Comparison of methods

To benchmark spatial domain segmentation performance, we compared GAADE with four state-of-the-art methods—DeepST [30], stLearn [31], SpaGCN [32], and SEDR [33]—using the DLPFC dataset. For slices 151669, 151670, 151671, and 151,672, the target number of clusters was set to 5, while for other sections, the target was set to 7. All methods were utilized with their default parameter settings.

To evaluate SVG identification, we quantified spatial autocorrelation using Moran’s I and Geary’s C for SVGs detected by GAADE and five other methods: SpatialDE, SpaGCN, Squidpy [34], scGCO [13] and SPARK. We filtered out cells with mitochondrial gene expression ratios above 15%, genes detected in fewer than three cells and low-expression cells with fewer than 100 detected genes. Gene expression levels were log-normalized following each method’s default settings prior to SVG detection.

**Figure 2:**
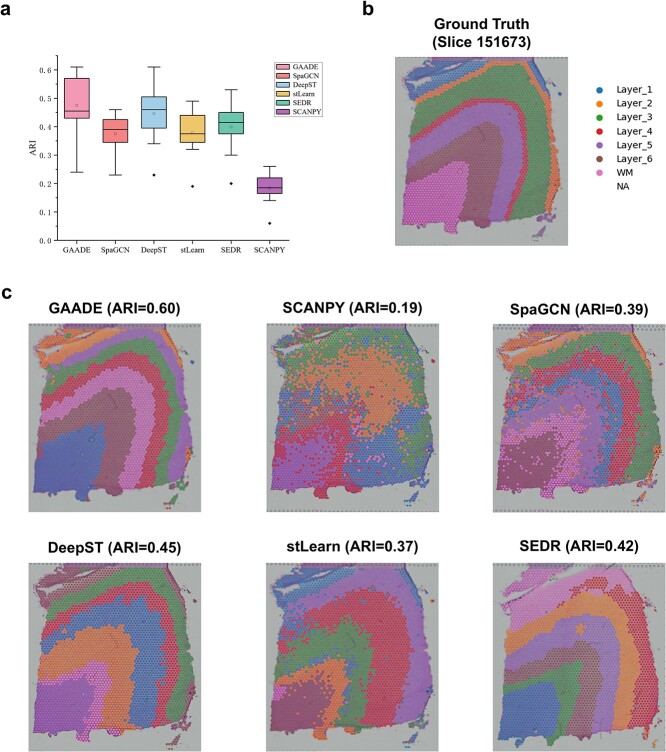
Identification of spatial domains in the LIBD dorsolateral prefrontal cortex data by GAADE. (a) Boxplot showing the clustering accuracy of GAADE and current state-of-the-art algorithms (SpaGCN, DeepST, stLearn, SEDR, and SCANPY) across all 12 sections of the DLPFC dataset, as measured by the ARI scores. (b) on slice 151,673, the actual locations of points are mapped to their spatial positions, which are divided into six cortical layers (L1-L6) and white matter (WM). (c) Clustering assignments generated by GAADE, SCANPY, SpaGCN, DeepST, stLearn, and SEDR in the DLPFC slice 151,673.

## Results

### Evaluation of GAADE’s performance in spatial domain segmentation

To quantitatively evaluate the spatial clustering performance of GAADE, we conducted tests on the DLPFC dataset, which consists of 12 slices depicting four- or six-layer structures of the human dorsolateral prefrontal cortex and white matter (WM). We compared GAADE against the non-spatial clustering method SCANPY and four recently developed spatial clustering methods: DeepST, stLearn, SpaGCN, and SEDR, using the Adjusted Rand Index (ARI) as a metric. Results indicate that the spatial domains identified by GAADE largely correspond to the manually annotated DLPFC structures and the cortical layer definitions in neuroscience.

In contrast, the non-spatial method SCANPY failed to clearly identify the layer structures in tissue slices. While existing spatial methods could detect most visible domains, they still struggled to achieve the precision of manual annotations in defining domain boundaries, with ARI scores generally lower than those of GAADE. GAADE demonstrated greater sensitivity and more consistent performance across all 12 slices (Fig. 2a). Through a detailed comparison of these methods, we found that the four spatial clustering algorithms that utilize spatial information outperform non-spatial clustering algorithms, confirming the performance improvements from integrating spatial information (Supplementary Figs. 1–11). Specifically, GAADE achieved an average ARI score of 0.475, significantly higher than the currently best method for spatial domain segmentation, DeepST (average ARI score = 0.45). In contrast, the SpaGCN method, which does not rely on spatial arrangement of points, obtained the lowest average ARI score of 0.375, with stLearn at an average ARI of 0.378, SEDR at 0.404, and the non-spatial clustering algorithm SCANPY scoring the lowest at only 0.184. Additionally, GAADE’s median ARI score was 0.455, second only to DeepST (median ARI score = 0.475). Other peer-reviewed methods’ median ARI scores were as follows: SEDR at 0.411, SpaGCN at 0.39, stLearn at 0.375 and SCANPY at 0.185. Both DeepST and SEDR showed significant performance variability across different slices. SpaGCN and stLearn had less variance in their ARI scores but poorer median scores (Table 2). These findings collectively indicate that GAADE possesses the best capability in identifying the spatial domain structure within the DLPFC dataset.

**Table 2:** ARI scores for all 12 slices in the DLPFC dataset.

Dataset	GAADE	SpaGCN	DeepST	stLearn	SEDR	SCANPY
DLPFC_151507	0.50	0.43	0.49	0.49	0.39	0.22
DLPFC_151508	0.44	0.34	0.43	0.47	0.36	0.15
DLPFC_151509	0.46	0.42	0.37	0.44	0.41	0.19
DLPFC_151510	0.43	0.42	0.47	0.44	0.39	0.14
DLPFC_151,669	0.24	0.23	0.34	0.32	0.30	0.06
DLPFC_151,670	0.45	0.35	0.23	0.19	0.20	0.18
DLPFC_151,671	0.58	0.46	0.48	0.38	0.44	0.22
DLPFC_151,672	0.56	0.39	0.42	0.34	0.46	0.18
DLPFC_151,673	0.60	0.39	0.50	0.37	0.42	0.19
DLPFC_151,674	0.40	0.35	0.52	0.35	0.42	0.26
DLPFC_151675	0.61	0.43	0.61	0.38	0.53	0.18
DLPFC_151676	0.43	0.29	0.54	0.37	0.47	0.24
Mean	0.475	0.375	0.45	0.37	0.40	0.18

We further evaluated the spatial domain delineation performance of GAADE compared to other methods on the DLPFC dataset, using manual annotations by Maynard et al. [35], based on morphological features and gene markers, as a reference (Fig. 2b). For example, in DLPFC slice 151,673, the spatial domains identified by DeepST and SEDR showed a better alignment with the manually annotated tissue layers compared to SpaGCN (Fig. 2c). Even when integrating histological image information to supplement spatial data, SpaGCN achieved an ARI score of only 0.39, significantly lower than DeepST and SEDR (GAADE ARI: 0.60, DeepST ARI: 0.45, SEDR ARI: 0.42, stLearn ARI: 0.37, SCANPY ARI: 0.19). In contrast, GAADE accurately identified the structures of the WM and the adjacent cortical layers, showing a high concordance with the manual annotations.

Although nearly all spatial methods detected the seven visible structural domains within the tissue sections, existing spatial methods still exhibit some shortcomings. For example, SEDR incorrectly merged the first (L1) and second (L2) layers, and mistakenly assigned the spatial information of the third layer to the fourth. This may be due to the sparse cell density in the first layer, where both DeepST and stLearn showed considerable deviations from the manual annotations in their segmentation of the first layer’s spatial domain. SpaGCN, meanwhile, generated incorrect layer thickness and also failed to accurately capture the boundary between the sixth layer and the WM layer.

### Analysis of GAADE’s performance in SVGs identification

**Figure 3:**
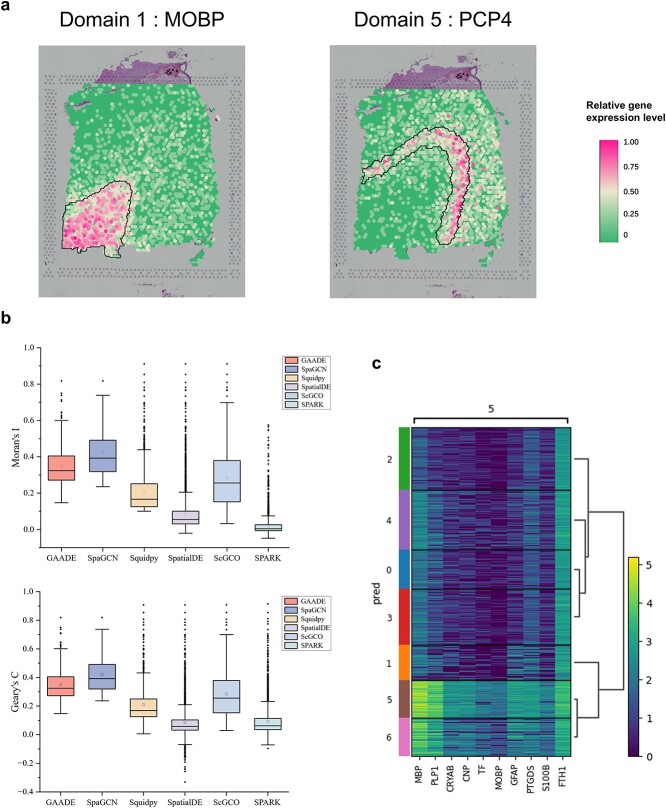
Performance of SVGs detected by GAADE in the LIBD dorsolateral prefrontal cortex data. (a) Spatial expression patterns of SVGs in spatial domains 1 (MOBP) and 5 (PCP4) in slice 151,673. (b) Boxplot of Moran’s I and Geary’s C values for SVGs detected by GAADE, SpaGCN, Squidpy, SpatialDE, ScGCO, and SPARK in slice 151,673. (c) High expression genes identified by the SpaGCN method in spatial domain 5 of the DLPFC 151673 slice data.

We conducted DE analysis by first selecting a target domain and considering the expression differences between the target domain and the first-order and second-order neighborhoods to identify region-specific genes. GAADE detected a total of 370 SVGs, with 108 specific SVGs identified in Domain 1, corresponding to the white matter layer. Domain 4 identified the highest number of 258 region-specific SVGs, with a substantial overlap of SVGs identified in other structural domains compared to those recognized in Domain 4. These results indicate that the gene expression differences between the six neuronal layers are minor, whereas the gene expression patterns in the white matter spots are distinctly different from those in the neuronal layers. Notably, GAADE delineated the boundary between the cortical layers and adjacent white matter (WM) in the tissue sections using WM/Oligodendrocyte specific gene MOBP and identified the marker gene PCP4 for layer L5 (Fig. 3a).

To assess GAADE’s performance in identifying SVGs, a comparison was made with five representative methods: SpatialDE, SpaGCN, Squidpy, scGCO, and SPARK. To evaluate the credibility of the SVGs detected by these methods, we quantitatively calculated the spatial autocorrelation coefficients Moran’s I and Geary’s C for the SVGs (Table 3).

**Table 3:** The average number of detected SVGs, the median of Moran’s I and the median of Geary’s C for all 12 slices in the DLPFC dataset using the methods GAADE, SpaGCN, Squidpy, SpatialDE, ScGCO, and SPARK.

	Number of SVGs	Median of Moran’s I	Median of Geary’s C
GAADE	138	0.3576	0.3579
SpaGCN	14	0.4747	0.0620
Squidpy	438	0.1644	0.4766
SpatialDE	1512	0.0518	0.1684
ScGCO	165	0.2115	0.2142
SPARK	982	0.1329	0.1478

In slice 151,673, GAADE identified a total of 370 useful SVGs. As a comparison, we verified that SpatialDE, without filtering for low-expression genes, recognized 3378 statistically significant SVGs, while SPARK and Squidpy identified 2951 and 855 SVGs, respectively. Although these three comparison methods have the advantage in the number of SVGs identified, the genes detected by these methods do not distinguish the varying degrees of spatial expression variation, as the SVGs identified by them show highly skewed adjusted p-values (FDR) towards 0 (Supplementary Fig. 23), with SpatialDE having 622 SVGs with an adjusted p-value of 0, SPARK having 1652 and Squidpy 557, and most of them only marked white matter regions. We also found that the SVGs detected by existing methods lack spatial domain specificity. Figure 3b shows that GAADE’s Moran’s I and Geary’s C are significantly higher than the genes detected by SpatialDE, SPARK, and Squidpy and are the highest among all methods except SpaGCN. Although SpaGCN’s median Moran’s I and Geary’s C values are slightly higher than our method GAADE (GAADE’s median Moran’s I is 0.3240, SpaGCN’s is 0.3923; GAADE’s median Geary’s C is 0.3245, SpaGCN’s is 0.392), SpaGCN detected only 60 SVGs (49 in spatial domain 5), whereas GAADE identified six times that number. Additionally, using the Wilcoxon rank-sum test, we analyzed the DE genes between spatial domains identified by SpaGCN. The results show that in spatial domain 5, which identified the most SVGs, the top ten genes with the highest expression levels are displayed through a heatmap (Fig. 3c). Simultaneously, GAADE detected all genes among the top ten highly expressed genes identified in spatial domain 5. Due to the lack of consideration for spatial domains, methods such as SpatialDE, Squidpy, scGCO, and SPARK often detect genes without a clear spatial expression pattern. Existing spatial domain-based methods for identifying SVGs, such as SpaGCN, predefine the similarity between adjacent spots prior to training, which hinders adaptive learning and limits generalizability across different tissues or samples. This approach may also lead to inaccurate identification of specific genes in boundary regions. GAADE not only identifies these SVGs with significant spatial expression patterns but also further optimizes the SVG filtering mechanism through DE analysis between the target domain and the first and second-order neighborhoods, thus increasing the number of SVGs identified. From these conclusions, we find that the GAADE model is better at capturing subtle expression differences in spatial domains and can better understand tissue cellular heterogeneity, intercellular communication and how cells respond to their microenvironment.

### Enhancing spatial domain segmentation and SVG identification performance with attention mechanisms

Building on previous discussions, we have designed an additional module for refining spatial domains specifically for ST data with low spatial resolution by incorporating attention mechanisms. By applying this module, we can better reveal the heterogeneous spatial similarities between adjacent points. We also employ the ARI to compare the performance of spatial domain identification within the DLPFC dataset. After introducing the attention mechanism, the GAADE model demonstrated improved spatial domain segmentation performance in more than half of the analyzed slices. Although the number of SVGs identified by the GAADE model decreased on some data, the introduction of the attention mechanism significantly increased both Moran’s I and Geary’s C in nine of the slices (Table 4). Further analysis indicates that while the introduction of attention mechanisms may result in a decrease in the number of SVGs in certain slices, this mechanism evidently aids in more effectively capturing spatial structures and distribution patterns, thereby enhancing identification accuracy.

**Table 4:** Comparison of ARI, median of Moran’s I and median of Geary’s C for GAADE before and after adding the attention mechanism.

Dataset	ARI		Median of Moran’s I		Median of Geary’s C	
	GAADE	GAADE_att	GAADE	GAADE_att	GAADE	GAADE_att
DLPFC_151507	0.50	0.56	0.3246	0.3542	0.3255	0.3543
DLPFC_151508	0.44	0.52	0.4131	0.3832	0.4130	0.3833
DLPFC_151509	0.46	0.47	0.2790	0.3169	0.2774	0.3211
DLPFC_151510	0.43	0.47	0.2289	0.2403	0.2288	0.2400
DLPFC_151,669	0.24	0.24	0.4194	0.4282	0.4207	0.4284
DLPFC_151,670	0.45	0.44	0.5401	0.5822	0.5404	0.5818
DLPFC_151,671	0.58	0.58	0.4673	0.4673	0.4685	0.4685
DLPFC_151,672	0.56	0.54	0.3654	0.3599	0.3660	0.3602
DLPFC_151,673	0.60	0.60	0.3240	0.3274	0.3245	0.3275
DLPFC_151,674	0.40	0.54	0.3169	0.3197	0.3169	0.3205
DLPFC_151675	0.61	0.65	0.3127	0.3275	0.3131	0.3277
DLPFC_151676	0.43	0.46	0.2998	0.3218	0.3003	0.3226

**Figure 4:**
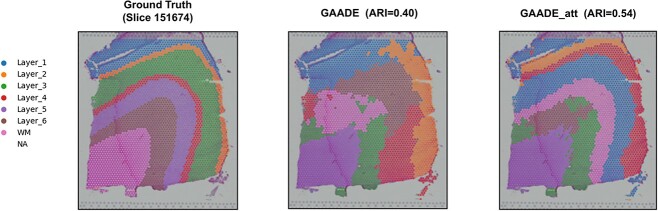
Spatial domains generated by GAADE in the LIBD dorsolateral prefrontal cortex data before and after the introduction of the attention mechanism.

Specifically, taking the 151,674 slice of the DLPFC dataset as an example, the introduction of an attention mechanism in the GAADE model significantly optimized the precise identification of spatial domains in this slice. The model with the added attention mechanism more clearly delineated layer boundaries and achieved optimal clustering accuracy (ARI = 0.54), compared to an ARI of only 0.40 without considering this module. Moreover, the GAADE model without the attention mechanism presented many outliers and could only correctly identify the white matter structure (Fig. 4). In contrast, the use of the refined spatial domain embedding module generally adhered to the expected layer patterns of this section, capturing the heterogeneity of the tissue structure more accurately. Additionally, after incorporating the refined spatial domain module, a significant increase in the ARI was observed in eight of the twelve slices (Supplementary Figs. 12–22). This indicates that the addition of the refined spatial domain enhances the capability of spatial domain identification. Although in some slices, the refinement module might lead to a reduction in the number of identified SVGs, overall, there was a significant improvement in the Moran’s I and Geary’s C metrics for the identified SVGs. The improvement in these metrics indicates that the refined spatial domain module can more accurately identify the spatially specific expression of genes. This capability is particularly important in complex tissue structures, where gene expression patterns are often more varied and intricate. Therefore, despite a reduction in the number of SVGs in some cases, the refined spatial domain module generally enhances the accuracy and quality of SVG identification, while also improving spatial domain segmentation performance in most instances.

### Identification of spatial domain specific SVGs without spatial prior information

**Figure 5:**
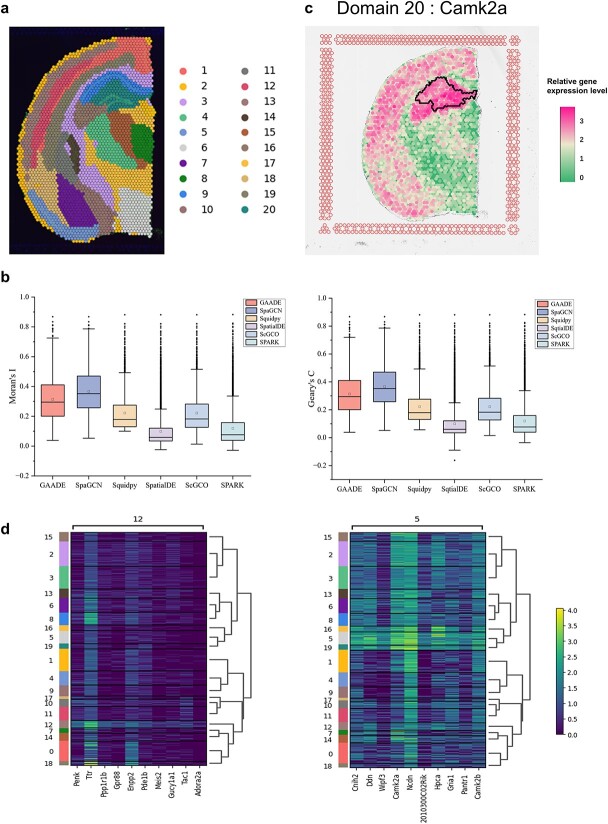
Spatial domains and SVGs detected in mouse coronal brain slice data. (a) Clustering results of GAADE for mouse coronal brain slices. (b) Boxplot of Moran’s I and Geary’s C values for SVGs detected by GAADE, SpaGCN, Squidpy, SpatialDE, ScGCO, and SPARK in mouse coronal brain slice data. (c) Spatial expression pattern of SVGs in mouse coronal brain spatial domain 20 (Camk2a). (d) High expression genes identified by the SpaGCN method in spatial domains 12 and 5 of mouse coronal brain slice data.

To quantitatively assess the spatial clustering performance of GAADE, We applied GAADE to three datasets from the 10X Visium ST platform, including 10 μm coronal sections of the adult mouse brain. We found that the clustering results identified by GAADE clearly delineated tissue structures containing different cell types and accurately recognized small spatial domains. Specifically, in the hippocampal area, GAADE identified several key structures such as the CA1 region (Domain 20) and CA3 (Domain 16) of Ammon’s horn (Fig. 5a).

Furthermore, we evaluated GAADE’s performance in identifying SVGs and compared it with five representative methods: SpatialDE, SpaGCN, Squidpy, scGCO, and SPARK. To assess the credibility of the SVGs detected by these methods, we also calculated the Moran’s I and Geary’s C metrics using the spatial information of SVGs. GAADE detected 1940 SVGs across twenty spatial domains, while SpatialDE, SPARK, Squidpy, and scGCO detected 8440, 6205, 2577 and 2380 SVGs, respectively (Table 5). As shown in Fig. 5b, although SPARK and SpatialDE detected a much higher number of SVGs than GAADE, the Moran’s I values of SVGs detected by GAADE were significantly higher than those detected by SPARK and SpatialDE. For Squidpy and scGCO, the number of SVGs detected was only slightly higher than those detected by GAADE, but their Moran’s I values were also lower than those detected by GAADE (median Moran’s I for GAADE is 0.2950, SpatialDE is 0.0587, SPARK is 0.0760, Squidpy is 0.1787, scGCO is 0.1819). The same trend is observed with the Geary’s C values of the SVGs detected by these methods. More stringent filtering of spots and genes did not improve the performance of these four models. Additionally, these four methods share a common issue regarding the spatial variability of gene expression, as their p-value distributions are highly skewed towards zero (Supplementary Fig. 24). However, due to the lack of consideration for tissue classification, the genes detected by these methods do not have guaranteed spatial expression patterns, making it challenging to use these genes for further biological research (this is also why the results of SVG detection are difficult to apply directly). In other words, these methods do not provide sufficient differentiation for the genes detected, failing to accurately reflect the degree of variation in spatial expression.

**Table 5:** Number of spatially variable genes (SVGs) identified by the five methods—SpatialDE, SpaGCN, Squidpy, scGCO, and SPARK—in different samples, along with the median values of Moran’s I and Geary’s C. (bold font indicates the optimal solution, while underlined text highlights the second-best solution.)

Dataset	Breast Cancer			Brain coronal			Brain (Sagittal-Anterior)		
	Number of SVGs	Median of Moran’s I	Median of Geary’s C	Number of SVGs	Median of Moran’s I	Median of Geary’s C	Number of SVGs	Median of Moran’s I	Median of Geary’s C
GAADE	1397	0.5428	0.5437	1940	0.2950	0.2946	2297	0.4041	0.4046
SpaGCN	642	0.6293	0.6300	789	0.3518	0.3521	445	0.5526	0.5523
Squidpy	2597	0.1508	0.1509	2577	0.1787	0.1794	4054	0.1833	0.1843
SpatialDE	12,363	0.0479	0.0484	8440	0.0587	0.0599	11,836	0.0666	0.0672
ScGCO	3168	0.1343	0.1344	2380	0.1819	0.1827	3585	0.1928	0.1929
SPARK	11,301	0.0519	0.0521	6205	0.0760	0.0767	9451	0.0809	0.0818

In contrast, GAADE’s use of multi-order neighborhood differential analysis filtering criteria can eliminate false positives in spatial gene variation, while ensuring that all detected SVGs have distinct spatial expression patterns. To illustrate the advantages of its method, take Domain 20 as an example. For each domain, GAADE identified SVGs that are enriched in that area. For instance, the marker gene Nptx1 was exclusively localized to the somata of CA3 pyramidal cells. Additionally, in Domain 20, we detected a total of 225 SVGs, with Camk2a being enriched in Domain 20 (Fig. 5c). In the hippocampus, the distribution pattern of Camk2a is distinct, highlighting the targeted dendritic area. Identifying region-specific distribution and dendritic localization may aid in the identification of conserved sequence elements associated with cell-specific and intracellular transport specificity.

Although SVGs detected by SpaGCN exhibited higher Moran’s I and Geary’s C values, the number of SVGs identified by SpaGCN was relatively few. To further assess the expression differences of these genes across different spatial domains, we employed the Wilcoxon rank-sum test to analyze DE genes between spatial domains identified by SpaGCN. Using heatmaps for visualization, we displayed the expression levels of these genes in spatial domains 12 and 5 (the two spatial domains where SpaGCN detected the highest number of SVGs), revealing the top ten characteristic genes of different spatial domains or cell types [36]. Further analysis revealed that of the top 20 highly expressed genes identified in these two domains, only one gene was not detected in GAADE. This indicates that GAADE not only identifies genes with spatial expression patterns detected by SpaGCN but also detects a larger number of SVGs (Fig. 5d).

**Figure 6:**
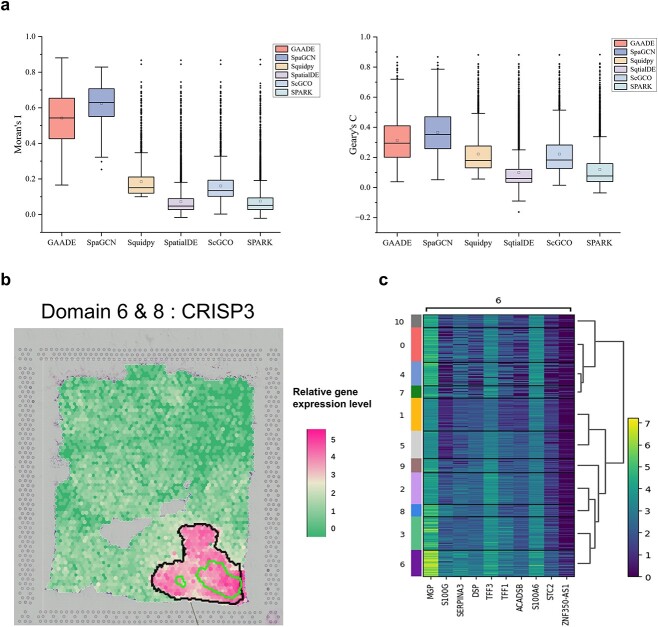
Analysis of SVGs detected in ductal carcinoma in situ slice data of human breast tissue. (a) Boxplot of Moran’s I and Geary’s C values for SVGs detected by GAADE, SpaGCN, Squidpy, SpatialDE, ScGCO, and SPARK in ductal carcinoma in situ slice data of human breast tissue. (b) Spatial expression patterns of SVGs in spatial domains 6 and 8 of ductal carcinoma in situ slices of human breast tissue (In the lower right corner of Figure 6, the outer annulus region is distinctly annotated as spatial domain 8, whereas the inner annulus area is precisely labeled as spatial domain 6). (c) Heatmap summarizing the high-expression genes identified by the SpaGCN method in spatial domain 6.

We analyzed a 5 μm ductal carcinoma in situ slice of FFPE human breast tissue using ST technology. Dimension reduction of the Visium data revealed 11 spatial clusters and GAADE identified 1397 SVGs. GAADE showed a clear advantage, with more distinct spatial domain patterns than other methods. In contrast, SpatialDE detected 12,363 SVGs (4210 with an FDR p-value of 0), SPARK identified 11 301 SVGs (7667 with an FDR-adjusted p-value of 0) and scGCO and Squidpy detected 3168 and 2597 SVGs, respectively, but with lower Moran’s I and Geary’s C values compared to GAADE (median Moran’s I: 0.5428 for GAADE, 0.1343 for scGCO and 0.1508 for Squidpy) (Fig. 6a).

Eight clusters were annotated with disease states, while three showed mixed cellular compositions. Spatial domains 6 and 8, forming the fibrotic region of invasive cancer, had the highest number of SVGs (223 and 242, respectively), with significant expression of CRISP3, a protein potentially influencing the tumor microenvironment and promoting cancer invasion (Fig. 6b). Although SpaGCN had slightly higher Moran’s I and Geary’s C values, it identified only 642 SVGs, limiting its usefulness in downstream analysis due to insufficient separation of cell types within the tissue. DE analysis using the Wilcoxon rank-sum test confirmed that GAADE could also identify genes with spatial expression patterns detected by SpaGCN (Fig. 6c).

**Figure 7:**
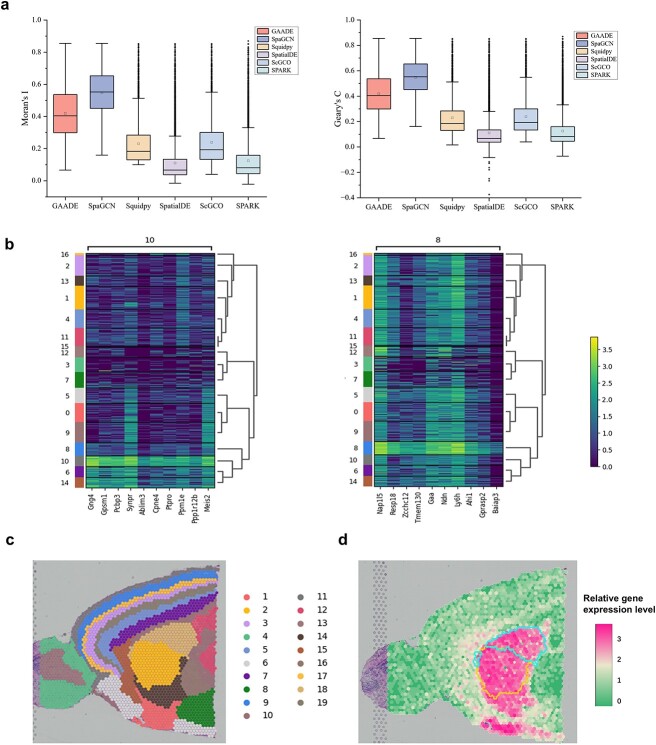
Spatial domains and SVGs detected in the anterior sagittal slice data of mouse brain tissue. (a) Boxplot of Moran’s I and Geary’s C values for SVGs detected by GAADE, SpaGCN, Squidpy, SpatialDE, ScGCO, and SPARK in anterior sagittal slice data of mouse brain tissue. (b) High expression genes identified by the SpaGCN method in spatial domains 10 and 8 of mouse sagittal brain slice data. (c) Clustering results of GAADE for the anterior brain tissue slices. (d) Spatial expression patterns of SVGs in spatial domains 17 and 18 of the anterior sagittal slices of mouse brain tissue (In the central region of the figure, the upper half is precisely annotated to signify Spatial Domain 18, while the lower half is distinctly labeled to denote Spatial Domain 17).

We analyzed the anterior sagittal slice dataset of mouse brain tissue, which includes 21,334 genes across 2695 locations. This dataset has a more complex tissue structure compared to the previous ones. We compared GAADE’s ability to detect SVGs with that of SpatialDE, SpaGCN, Squidpy, scGCO, and SPARK. With the number of clusters set to 19, GAADE identified 2297 SVGs across 19 spatial domains. In contrast, SPARK and SpatialDE detected 9451 and 11 836 SVGs, respectively, but these included noise. The median Moran’s I and Geary’s C values for SVGs detected by GAADE (Moran’s I: 0.4041) were significantly higher than those detected by SPARK (0.0809) and SpatialDE (0.0666) (Fig. 7a).

While Squidpy and scGCO identified 4064 and 3585 SVGs, respectively, many had an adjusted p-value of 0, making it difficult to assess spatial heterogeneity. SpaGCN had the highest median Moran’s I score (0.5526) but identified only 445 SVGs. GAADE, by considering inter-domain differential analysis and examining cell numbers and gene expression differences between target domains and second-order neighborhoods, detected 2297 SVGs with a median Moran’s I of 0.4041. While the number of SVGs does not always indicate accuracy, a higher number of SVGs with similar Moran’s I and Geary’s C values may better capture the complexity of spatial gene expression.

To evaluate expression differences across spatial domains, we applied the Wilcoxon rank-sum test to identify differentially expressed genes between domains detected by SpaGCN, highlighting the top ten characteristic genes for each domain or cell type. Notably, for the two domains where SpaGCN detected the highest number of SVGs, GAADE successfully identified all of the top 20 highly expressed genes, demonstrating its high sensitivity and coverage in detecting genes with spatial expression patterns (Fig. 7b).

Moreover, GAADE effectively delineates the histological structure of this complex region, clearly defining tissue boundaries. As shown in Fig. 7c, the identified spatial domains 2, 3, 5, 7, 9, 11, and 19 are marked according to their location in the anterior cortical layers of the mouse brain. GAADE also recognized well-known marker genes, including Camk2n1, Nrgn, and Atp1a1, as indicators of these domains. Further validation of domain-specific marker genes showed that Meis2 is enriched in Domain 13, while Ppp1r1b is highly expressed in Domains 17 and 18, suggesting their association with dopamine secretion and neural development (Fig. 7d). GAADE’s robust detection method ensures the accurate identification of SVGs, underscoring its superior capability in recognizing true spatial gene expression patterns.

## Conclusions

The rapid advancement of ST has revolutionized the study of spatial heterogeneity, providing new perspectives for analyzing cellular transcriptomes. The identification of SVGs is a critical step in characterizing spatial domains. In this study, we propose the GAADE framework, which integrates spatial location and gene expression data to model spatial embeddings, enabling the identification of spatial domains with similar expression patterns and the detection of SVGs with distinct spatial expression profiles.

GAADE first constructs a SNG to generate node representations and aggregates neighboring nodes through a stacked graph structure, clustering them into the same spatial domain. We conducted systematic tests across datasets from various species and tissues. When applied to the human DLPFC dataset, GAADE achieved optimal ARI values in over half of the 12 slices analyzed. The framework is flexible, allowing users to adjust parameters through a self-attention mechanism to prioritize either spatial domain identification or SVG quality. Upon incorporating a refined spatial domain boundary module, GAADE outperformed other models in spatial clustering, despite a reduction in the number of detected SVGs. However, the module significantly improved the Moran’s I and Geary’s C metrics for most SVGs, indicating higher consistency and significance in spatial distribution [37]. Thus, researchers should weigh the benefits of using this module according to different experimental requirements.

We focus our research on detecting SVGs within spatial domains. Most state-of-the-art SVG identification algorithms, such as nnSVG [14], do not consider spatial domain information, which limits the ability to identify genes with spatial-specific expression in specific micro-functional areas within tissues. Since there is a correlation between gene variability and expression levels, we do not consider the identification of spatial domains and SVGs as separate issues. By limiting the search space within precisely defined spatial domains, GAADE rigorously considers intra-domain gene expression and inter-domain differential analysis, and further analyzes the differences in cell numbers and gene expression levels between target domains and second-order neighborhoods, ensuring that the detected SVGs have definite spatial expression patterns. Comparisons using the Moran’s I spatial autocorrelation statistic show that GAADE is more capable of detecting SVGs with clearer spatial expression patterns than SpatialDE, Squidpy, scGCO, and SPARK. One limitation of GAADE is that it does not fully utilize histological image information. Future improvements could involve directly integrating histological images and spatial gene expression profiles to determine tissue structural domains, making the detected SVGs more biologically meaningful.

**Key Points**

- GAADE employs a graph attention autoencoder, selectively integrating gene expression and spatial location information to construct an accurate spatial neighbor network, thereby better representing the spatial similarities across different spatial domain boundaries. By restricting the search space to optimized spatial domains, this framework enhances the consistency and significance of the identified SVGs in their spatial distribution.
- By optimizing the identification of spatial domains, GAADE for the first time considers the expression differences between target spatial domains and their second-order neighborhoods, demonstrating enhanced performance in both the number of SVGs detected and the precision of capturing spatial variations in gene expression.
- GAADE utilizes a self-attention mechanism that enables users to selectively focus on either the identification of spatial domains or the quality of SVG detection by adjusting parameter configurations.

## Supplementary Material

Supplemental_Material_bribio_bbae669

## Data Availability

The source code and datasets of GAADE have been uploaded to https://github.com/Nefu-Hao/GAADE.
