# Supplementary material for: GAADE: identification spatially variable genes based on adaptive graph attention network
Source: Brief Bioinform. 2024 Dec 20;26(1):bbae669. doi: 10.1093/bib/bbae669 (PMC11658817; doi:10.1093/bib/bbae669)
Supplement: Supplemental_Material_bribio_bbae669 [file supplemental_material_bribio_bbae669.docx]

**Supplementary Materials**

**
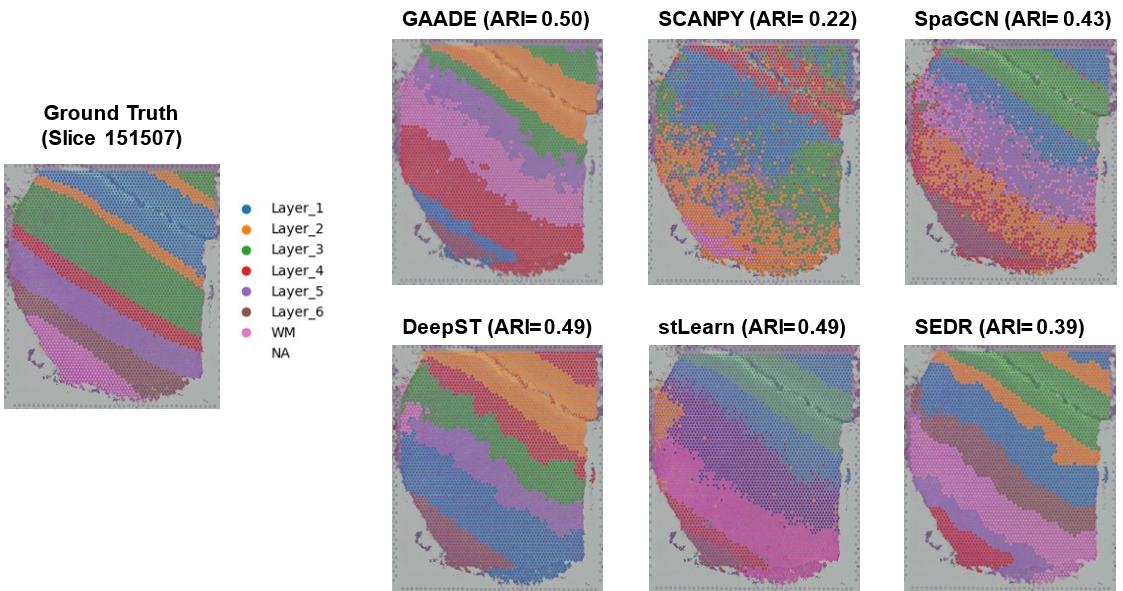
**

**Supplementary Figure 1.** Performance comparison of all algorithms on 151507 of DLPFCs. The spatial domain recognition results of all methods, including spatial algorithms (GAADE, SpaGCN, DeepST, stLearn, and SEDR) and non-spatial algorithms (SCANPY), on slice 151507 are presented. The leftmost figure shows the annotated dorsolateral prefrontal cortex (DLPFC) layers. The ground truth of spots was mapped to their spatial locations and divided into six cortical layers (L1-L6) and white matter (WM).


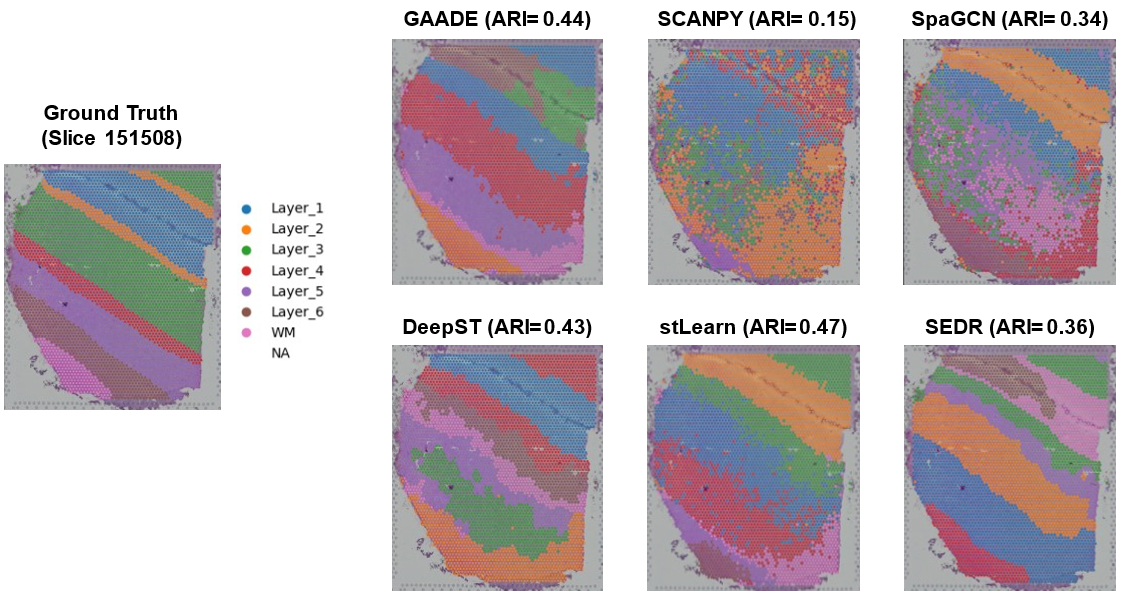


**Supplementary Figure 2.** Performance comparison of all algorithms on 151508 of DLPFCs. The spatial domain recognition results of all methods, including spatial algorithms (GAADE, SpaGCN, DeepST, stLearn, and SEDR) and non-spatial algorithms (SCANPY), on slice 151508 are presented. The leftmost figure shows the annotated DLPFC layers. The ground truth of spots was mapped to their spatial locations and divided into six cortical layers (L1-L6) and white matter (WM).


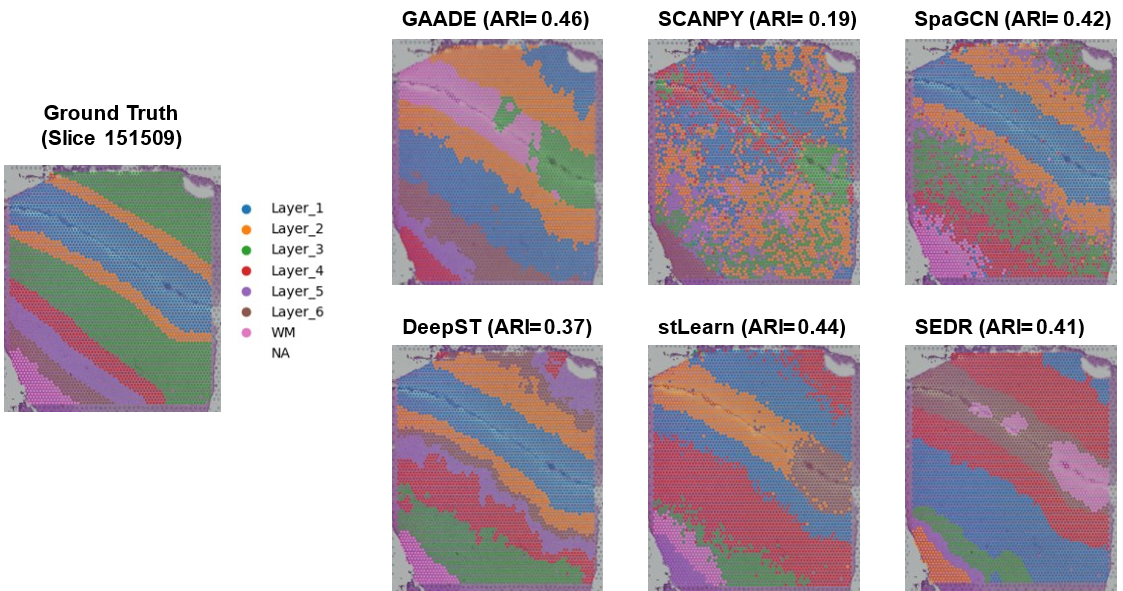


**Supplementary Figure 3.** Performance comparison of all algorithms on 151509 of DLPFCs. The spatial domain recognition results of all methods, including spatial algorithms (GAADE, SpaGCN, DeepST, stLearn, and SEDR) and non-spatial algorithms (SCANPY), on slice 151509 are presented. The leftmost figure shows the annotated DLPFC layers. The ground truth of spots was mapped to their spatial locations and divided into six cortical layers (L1-L6) and white matter (WM).


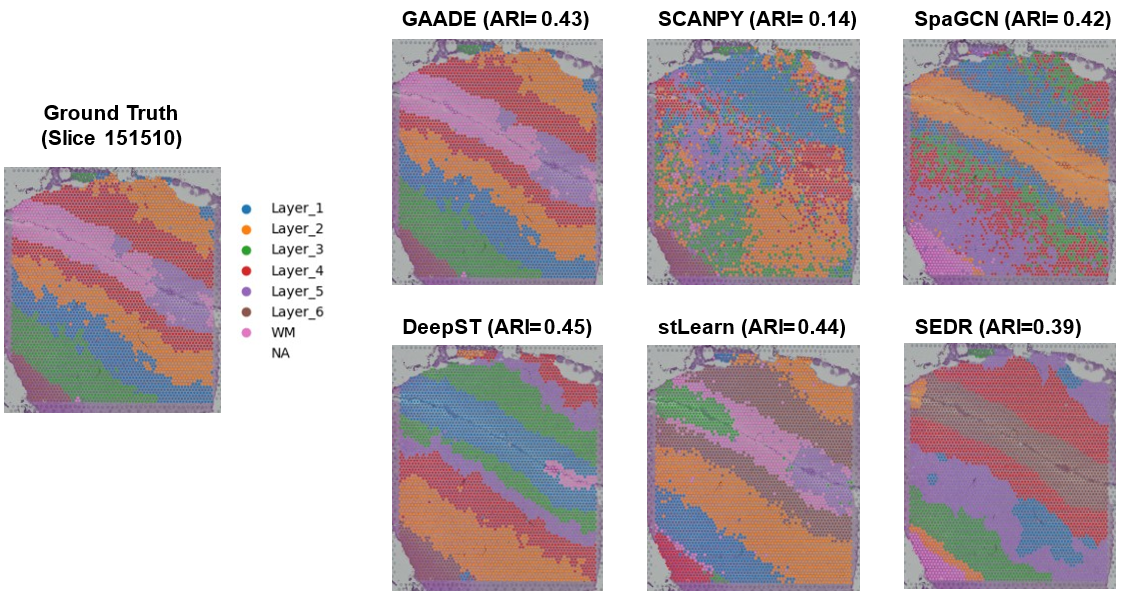


**Supplementary Figure 4.** Performance comparison of all algorithms on 151510 of DLPFCs. The spatial domain recognition results of all methods, including spatial algorithms (GAADE, SpaGCN, DeepST, stLearn, and SEDR) and non-spatial algorithms (SCANPY), on slice 151510 are presented. The leftmost figure shows the annotated DLPFC layers. The ground truth of spots was mapped to their spatial locations and divided into six cortical layers (L1-L6) and white matter (WM).


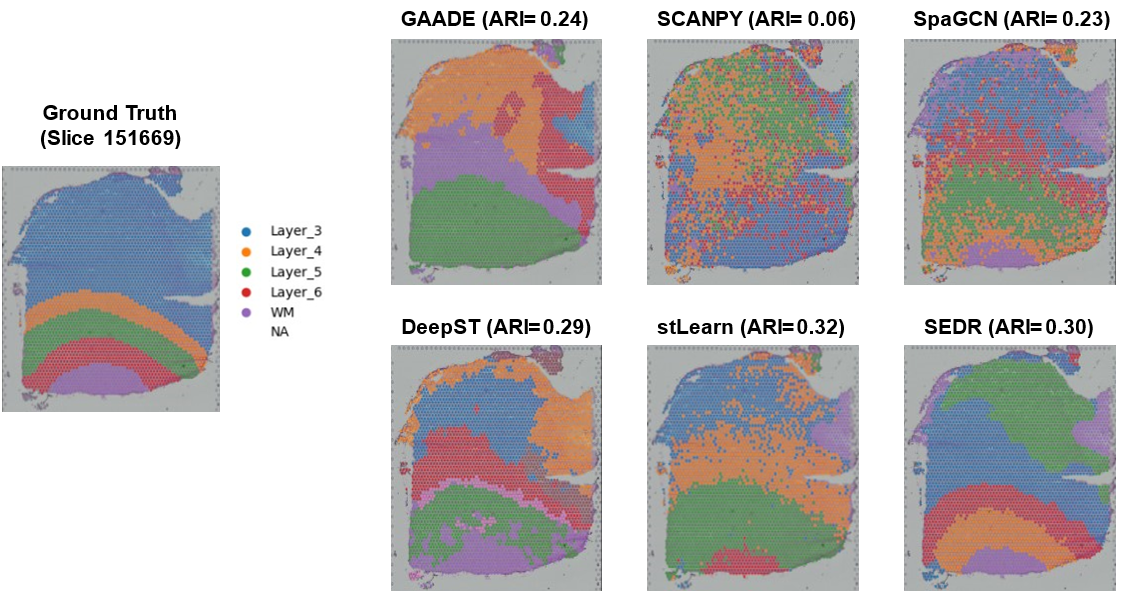


**Supplementary Figure 5.** Performance comparison of all algorithms on 151669 of DLPFCs. The spatial domain recognition results of all methods, including spatial algorithms (GAADE, SpaGCN, DeepST, stLearn, and SEDR) and non-spatial algorithms (SCANPY), on slice 151669 are presented. The leftmost figure shows the annotated DLPFC layers. The ground truth of spots was mapped to their spatial locations and divided into six cortical layers (L3-L6) and white matter (WN) layer.


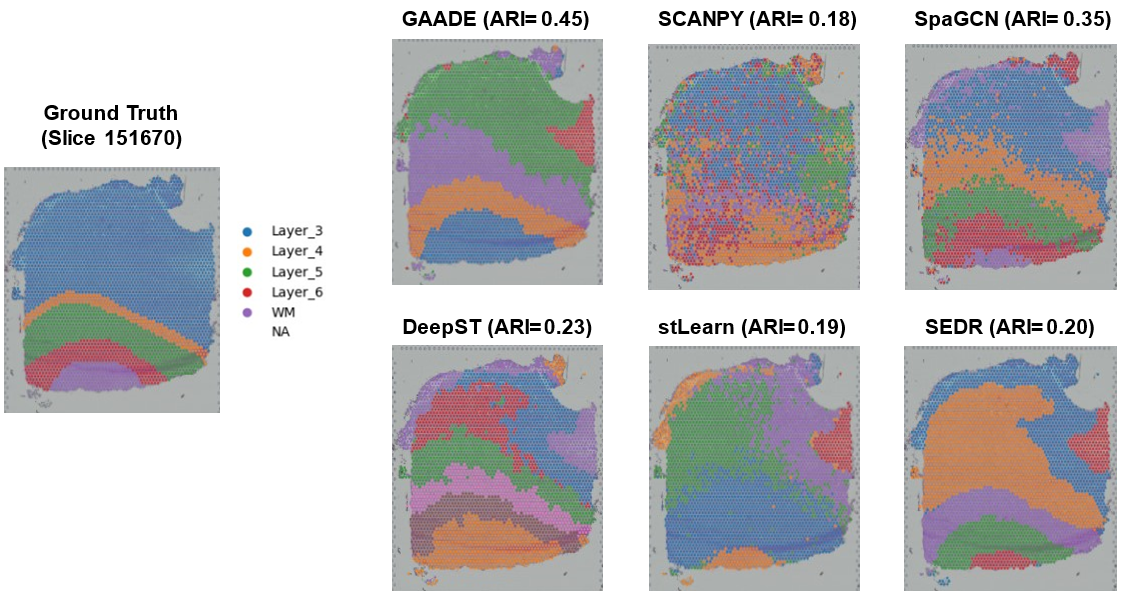


**Supplementary Figure 6.** Performance comparison of all algorithms on 151670 of DLPFCs. The spatial domain recognition results of all methods, including spatial algorithms (GAADE, SpaGCN, DeepST, stLearn, and SEDR) and non-spatial algorithms (SCANPY), on slice 151670 are presented. The leftmost figure shows the annotated DLPFC layers. The ground truth of spots was mapped to their spatial locations and divided into six cortical layers (L3-L6) and white matter (WN) layer.


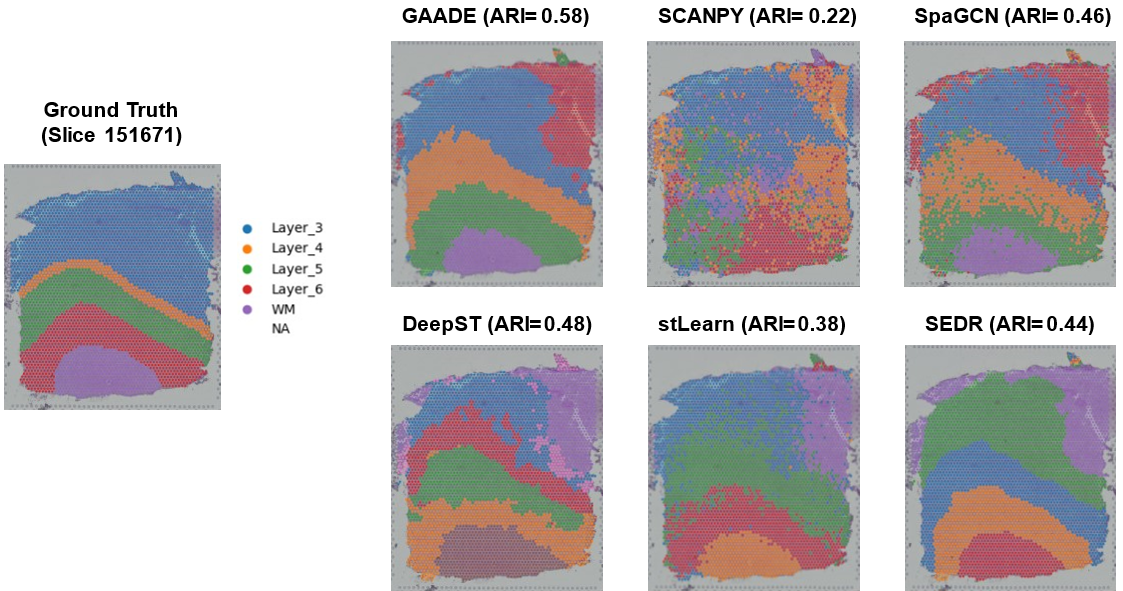


**Supplementary Figure 7.** Performance comparison of all algorithms on 151671 of DLPFCs. The spatial domain recognition results of all methods, including spatial algorithms (GAADE, SpaGCN, DeepST, stLearn, and SEDR) and non-spatial algorithms (SCANPY), on slice 151671 are presented. The leftmost figure shows the annotated DLPFC layers. The ground truth of spots was mapped to their spatial locations and divided into six cortical layers (L3-L6) and white matter (WN) layer.


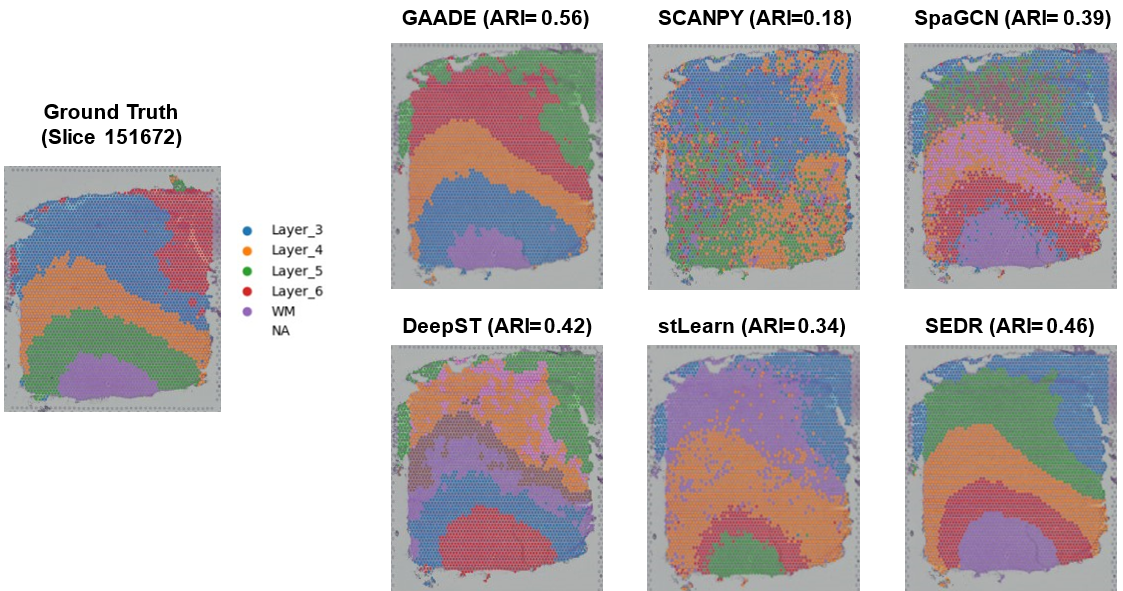


**Supplementary Figure 8.** Performance comparison of all algorithms on 151672 of DLPFCs. The spatial domain recognition results of all methods, including spatial algorithms (GAADE, SpaGCN, DeepST, stLearn, and SEDR) and non-spatial algorithms (SCANPY), on slice 151672 are presented. The leftmost figure shows the annotated DLPFC layers. The ground truth of spots was mapped to their spatial locations and divided into six cortical layers (L3-L6) and white matter (WN) layer.


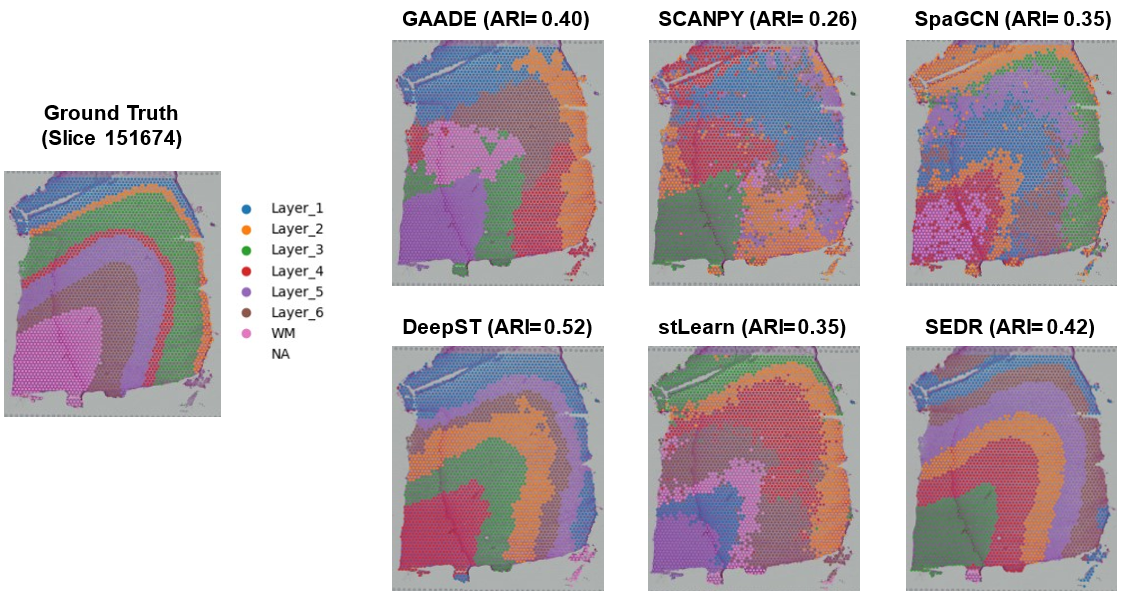


**Supplementary Figure 9.** Performance comparison of all algorithms on 151674 of DLPFCs. The spatial domain recognition results of all methods, including spatial algorithms (GAADE, SpaGCN, DeepST, stLearn, and SEDR) and non-spatial algorithms (SCANPY), on slice 151674 are presented. The leftmost figure shows the annotated DLPFC layers. The ground truth of spots was mapped to their spatial locations and divided into six cortical layers (L1-L6) and white matter (WM).


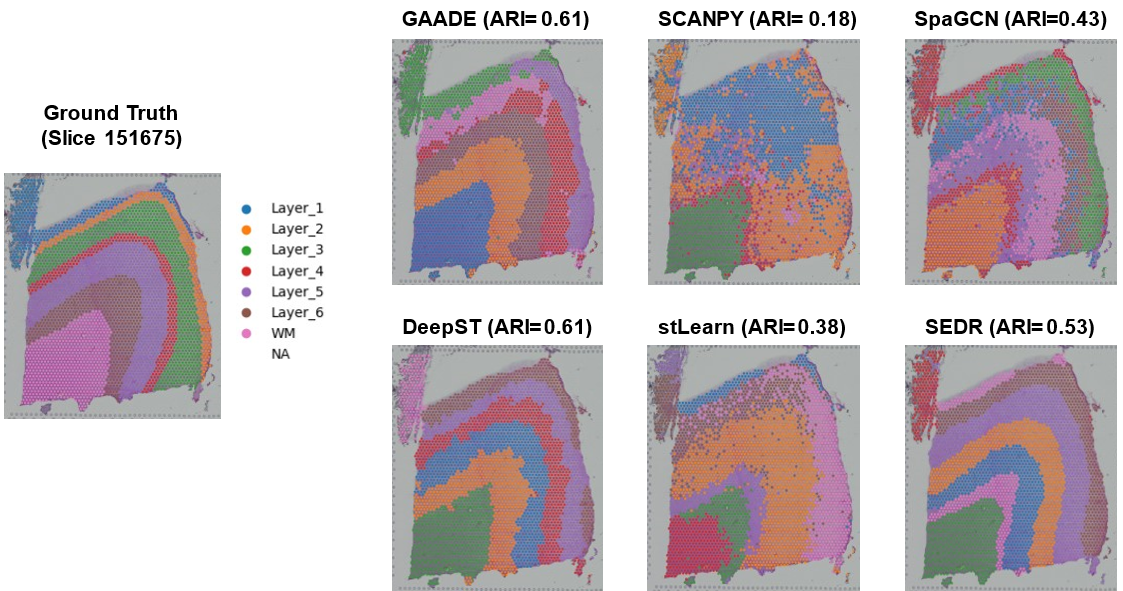


**Supplementary Figure 10.** Performance comparison of all algorithms on 151675 of DLPFCs. The spatial domain recognition results of all methods, including spatial algorithms (GAADE, SpaGCN, DeepST, stLearn, and SEDR) and non-spatial algorithms (SCANPY), on slice 151675 are presented. The leftmost figure shows the annotated DLPFC layers. The ground truth of spots was mapped to their spatial locations and divided into six cortical layers (L1-L6) and white matter (WM).


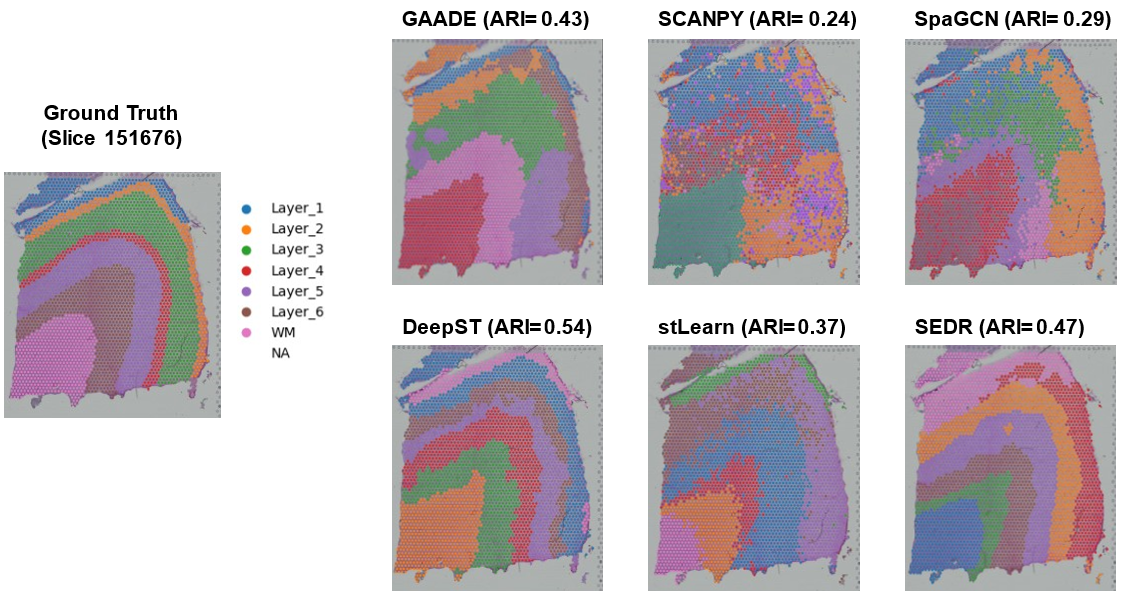


**Supplementary Figure 11.** Performance comparison of all algorithms on 151676 of DLPFCs. The spatial domain recognition results of all methods, including spatial algorithms (GAADE, SpaGCN, DeepST, stLearn, and SEDR) and non-spatial algorithms (SCANPY), on slice 151676 are presented. The leftmost figure shows the annotated DLPFC layers. The ground truth of spots was mapped to their spatial locations and divided into six cortical layers (L1-L6) and white matter (WM).


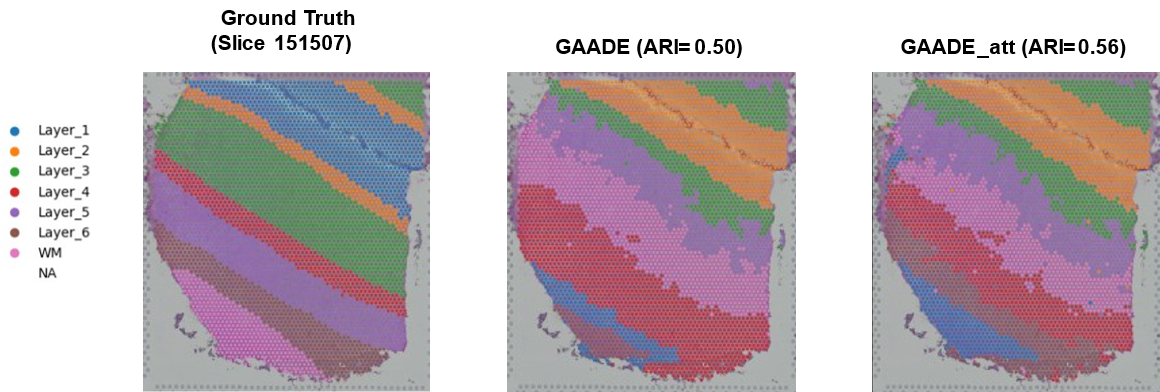


**Supplementary Figure 12.** Clustering results of spatial domains in the DLPFC section 151507 before and after the introduction of the attention mechanism. Results incorporating the attention mechanism are denoted as GAADE_att.


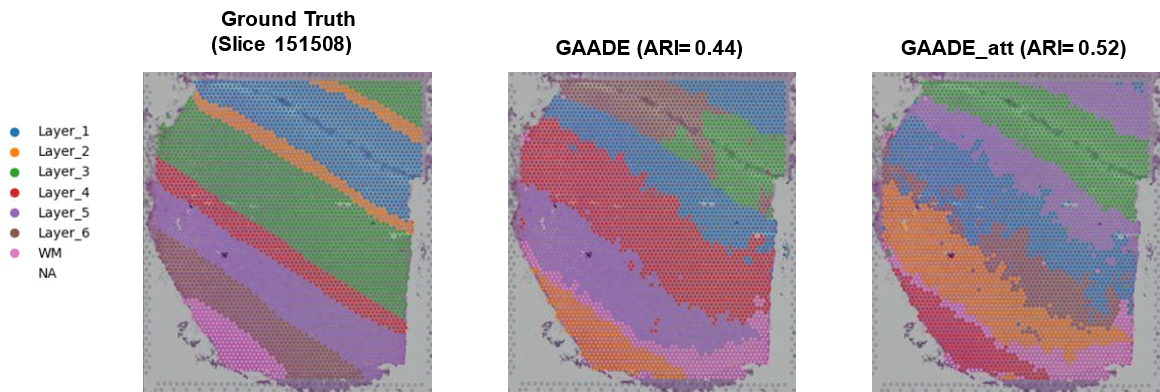


**Supplementary Figure 13.** Clustering results of spatial domains in the DLPFC section 151508 before and after the introduction of the attention mechanism. Results incorporating the attention mechanism are denoted as GAADE_att.


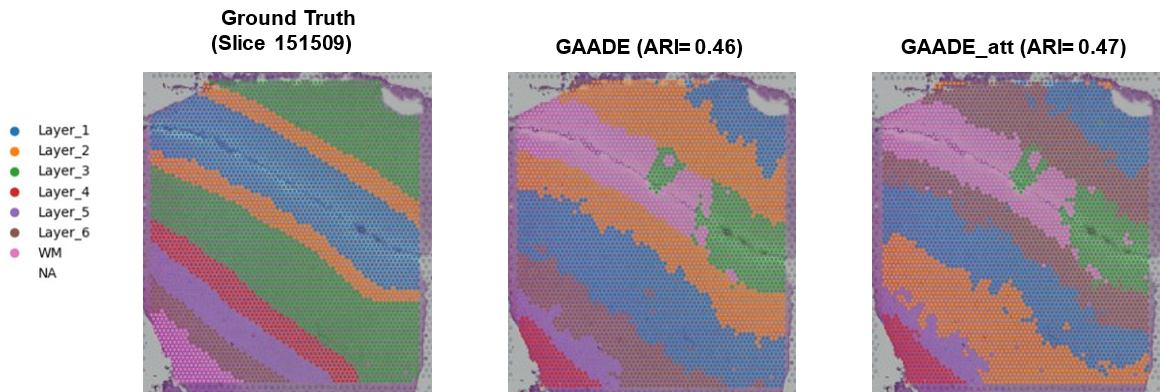


**Supplementary Figure 14.** Clustering results of spatial domains in the DLPFC section 151509 before and after the introduction of the attention mechanism. Results incorporating the attention mechanism are denoted as GAADE_att.


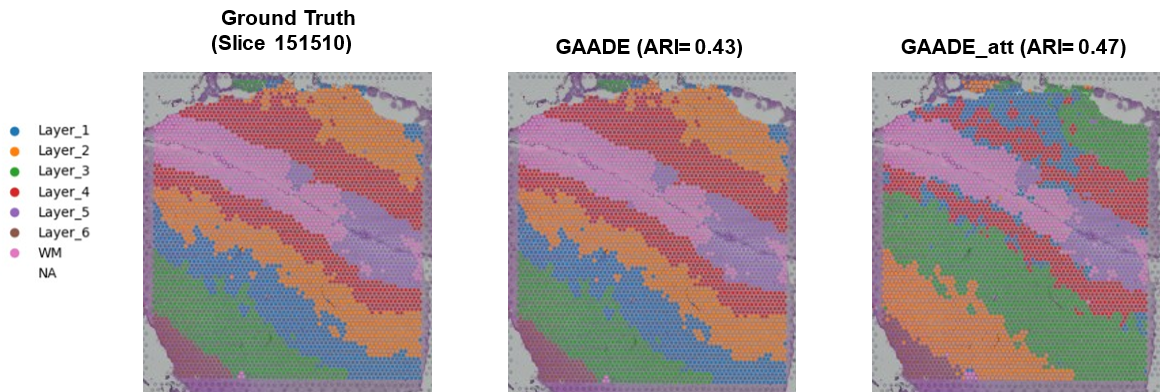


**Supplementary Figure 15.** Clustering results of spatial domains in the DLPFC section 151510 before and after the introduction of the attention mechanism. Results incorporating the attention mechanism are denoted as GAADE_att.


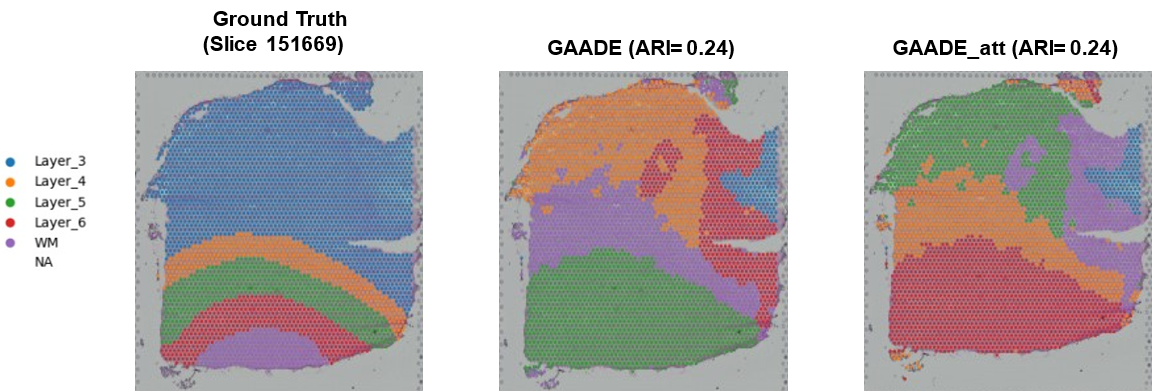


**Supplementary Figure 16.** Clustering results of spatial domains in the DLPFC section 151669 before and after the introduction of the attention mechanism. Results incorporating the attention mechanism are denoted as GAADE_att.


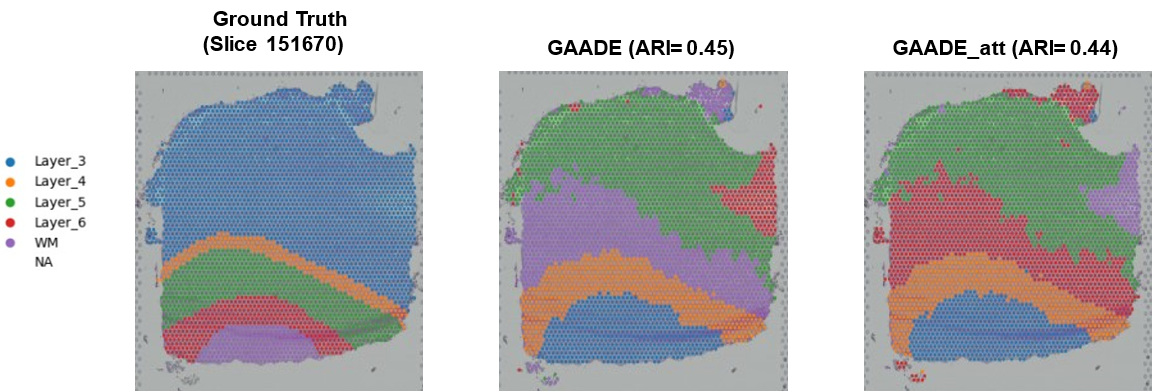


**Supplementary Figure 17.** Clustering results of spatial domains in the DLPFC section 151670 before and after the introduction of the attention mechanism. Results incorporating the attention mechanism are denoted as GAADE_att.


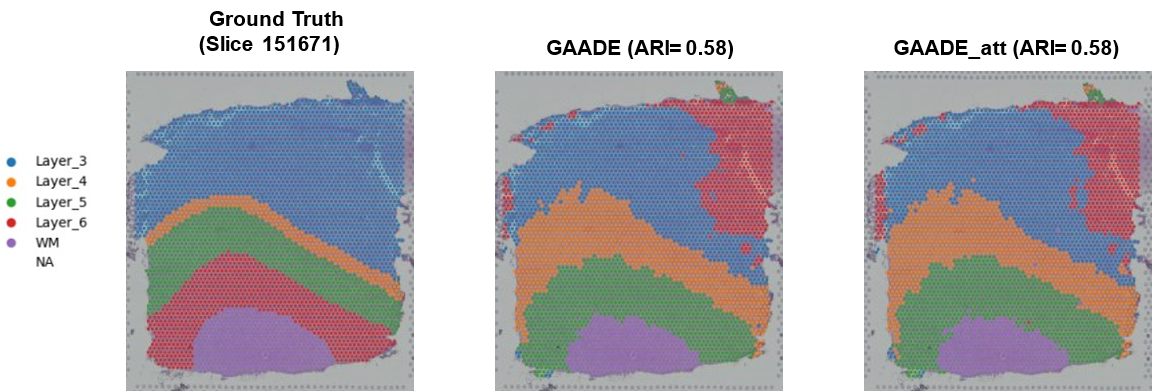


**Supplementary Figure 18.** Clustering results of spatial domains in the DLPFC section 151671 before and after the introduction of the attention mechanism. Results incorporating the attention mechanism are denoted as GAADE_att.


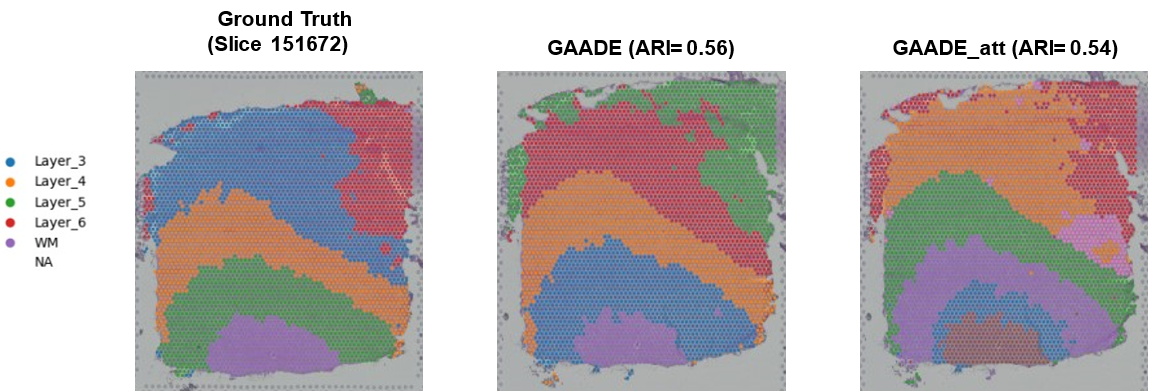


**Supplementary Figure 19.** Clustering results of spatial domains in the DLPFC section 151672 before and after the introduction of the attention mechanism. Results incorporating the attention mechanism are denoted as GAADE_att.


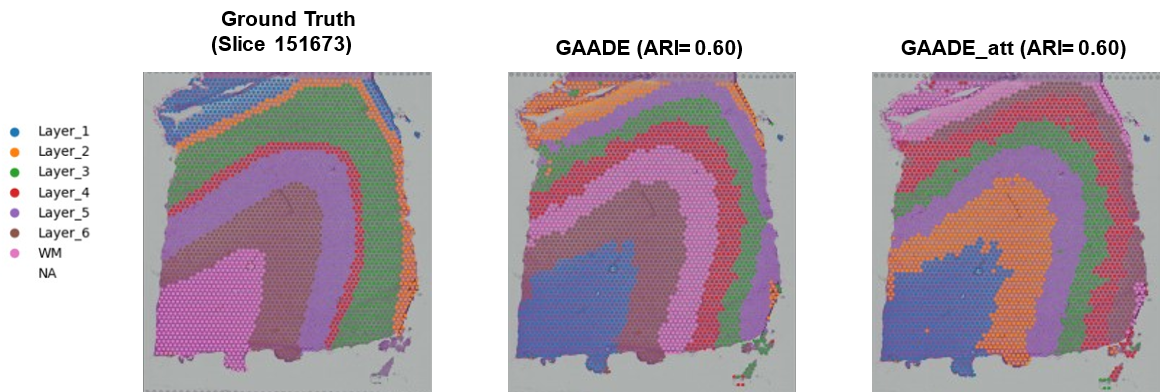


**Supplementary Figure 20.** Clustering results of spatial domains in the DLPFC section 151673 before and after the introduction of the attention mechanism. Results incorporating the attention mechanism are denoted as GAADE_att.


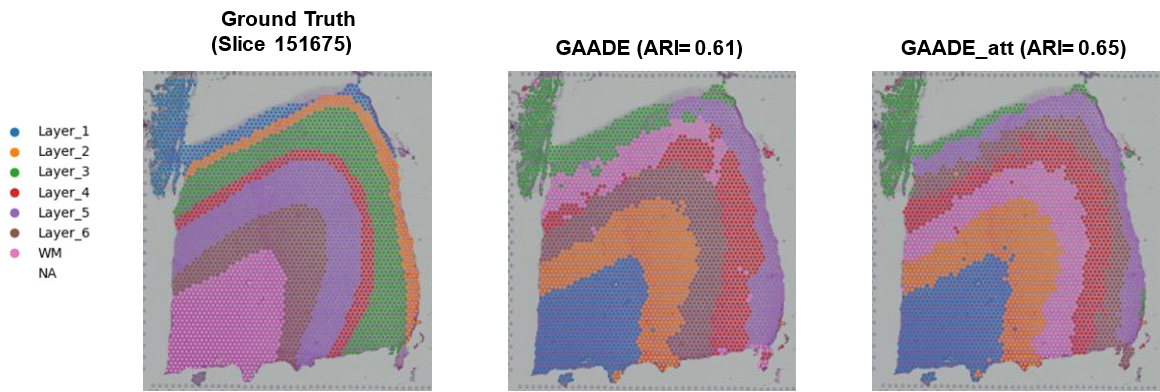


**Supplementary Figure 21.** Clustering results of spatial domains in the DLPFC section 151675 before and after the introduction of the attention mechanism. Results incorporating the attention mechanism are denoted as GAADE_att.


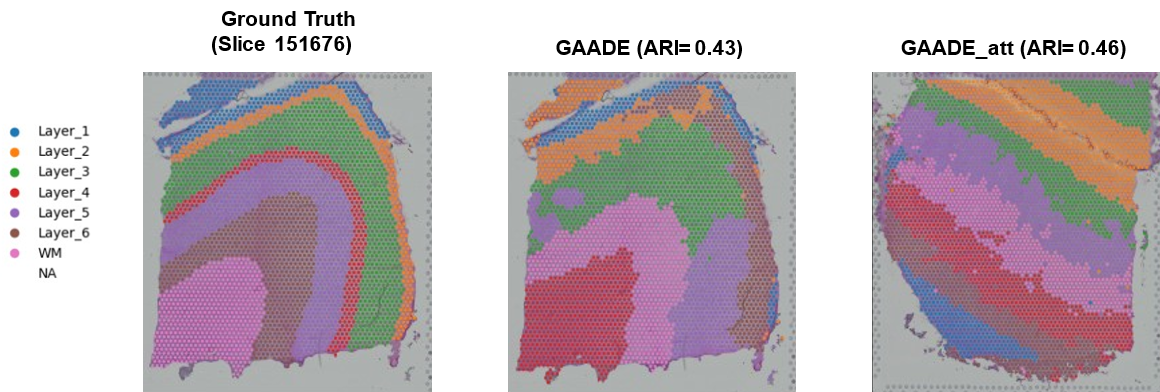


**Supplementary Figure 22.** Clustering results of spatial domains in the DLPFC section 151676 before and after the introduction of the attention mechanism. Results incorporating the attention mechanism are denoted as GAADE_att.


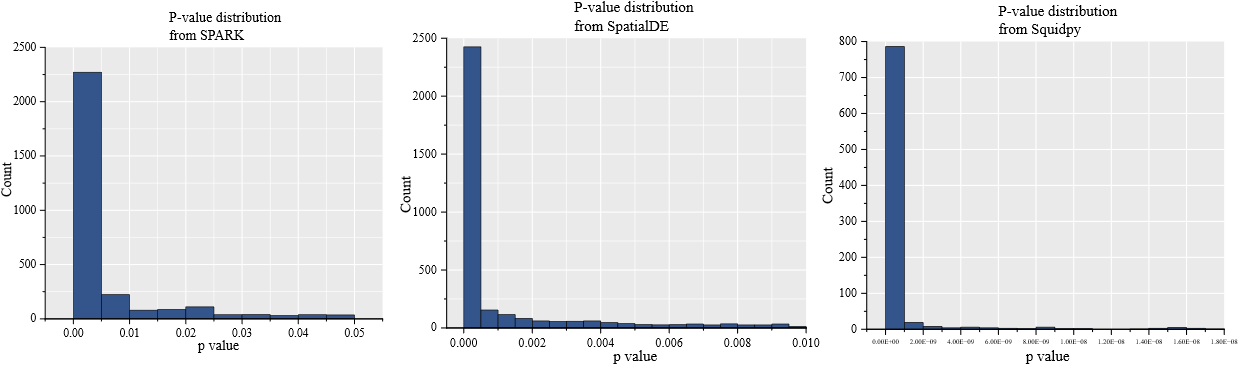


**Supplementary Figure 23.** Distribution of p-values (adjusted for FDR) of SVGs detected by SPARK, SpatialDE, and Squidpy in the LIBD human dorsolateral prefrontal cortex slice 151673. (The number of SVGs identified by scGCO is relatively small and therefore not included in the calculation).


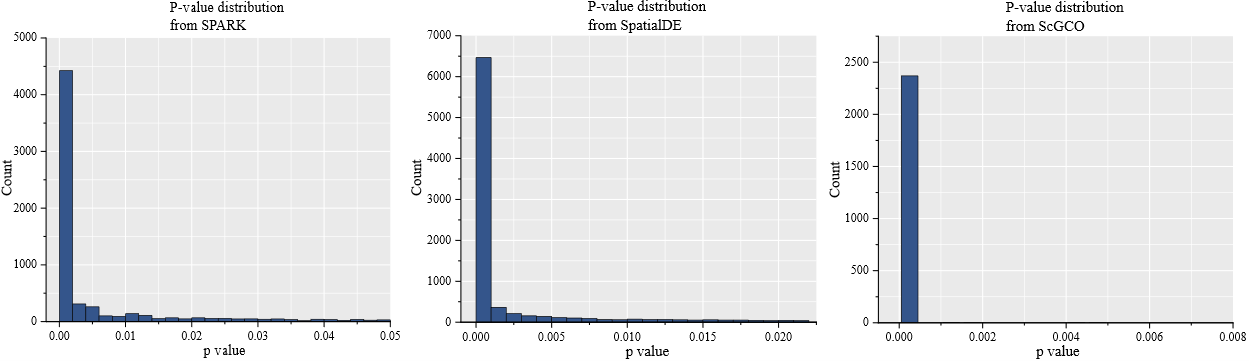


**Supplementary Figure 24.** Distribution of p-values (FDR-adjusted) of SVGs detected by SPARK, SpatialDE, and ScGCO in the mouse coronal brain slice data.( The p-values of SVGs detected by Squidpy are all zero and are therefore not shown in the figure.)
